# Continuous cropping of lavender (*Lavandula angustifolia Mill.*) enhances essential oil yield without compromising quality

**DOI:** 10.3389/fpls.2026.1840314

**Published:** 2026-05-29

**Authors:** Junnan Jian, Weikang Luo, Bian Ran, Ling Zhu, Shengjun Zhang, Xuechao Zhang, Jia Zeng, Shimin Tang, Shilei Dong

**Affiliations:** 1 Agricultural Science Institute of Ili Kazak Autonomous Prefecture, Yining, China; 2 Yili Perilla Beauty Biotechnology Co. Ltd., Yining, China; 3 College of Agronomy, Northwest A&F University, Yangling, Shaanxi, China

**Keywords:** lavender, continuous cropping, essential oil yield, bacteria, network complexity, sustainable agriculture

## Abstract

Yield decline due to continuous cropping poses a significant challenge to agricultural sustainability. However, systematic investigations into the effects of continuous cropping in lavender (*Lavandula angustifolia Mill.*) remain scarce. This study was conducted in the Yili River Valley, Xinjiang, the largest lavender-producing region in China. Three representative cultivation zones were selected. Continuous cropping duration gradients of 1, 3, and 6 years were established (with three replicates per treatment). We assessed essential oil quality and yield, soil physicochemical properties, enzyme activities, and bacterial community structure. Multivariate statistical analyses were employed to elucidate the “soil-microbe-plant” interaction mechanisms under lavender continuous cropping. Continuous cropping (6 years) significantly increased essential oil yield (+37.9%, *P* < 0.05) without compromising oil quality, as the relative contents of signature constituents (linalool, camphor, linalyl acetate, lavandulyl acetate) remained stable Soil nutrients exhibited a triphasic pattern (“low-high-low”). Organic carbon and total nitrogen peaked during the 3-year continuous cropping phase, primarily attributable to reduced soil disturbance under perennial lavender cultivation. Continuous cropping exerted a vertical stratification effect on soil bacterial α-diversity: no significant change in 0–20 cm topsoil (*P* > 0.05), but a significant decrease in 20–40 cm subsoil (*P* < 0.05). Bacterial co-occurrence network complexity decreased with cropping duration across all layers. Random Forest and Partial Least Squares Path Modeling (PLS-PM) analyses revealed that the synergistic effect of intensified microbial carbon limitation and alleviated nitrogen limitation was the core driver of increased oil yield. The reduced microbial network complexity under long-term continuous cropping implies potential soil health and long-term productivity risks, supporting a 3–5 year continuous cropping plus rotation strategy to balance short-term gains and long-term ecosystem health. This study clarifies the unique “stable quality-enhanced yield” effect in lavender continuous cropping and its regulatory mechanisms, elucidating the short-term-long-term trade-off to provide a theoretical framework and practical guidelines for sustainable aromatic crop production under continuous cropping systems.

## 1 Introduction

Lavender (*Lavandula angustifolia Mill.*), a term collectively referring to plants within the Lavandulagenus, is a perennial herb or subshrub renowned for its high ornamental value (Jackson et al., 2025). Owing to its essential oil rich in active compounds such as linalool and camphor, lavender also possesses significant commercial value in sectors including healthcare, cosmetics, and the food industry, establishing it as one of the world’s most important aromatic cash crops (Dong et al., 2024; Prusinowska et al., 2016). Benefiting from its unique climatic and edaphic conditions, the Yili River Valley in Xinjiang, China, has emerged as the largest lavender cultivation base in China. Influenced by existing cultivation practices, long-term continuous cropping is prevalent across the main lavender-producing areas in this region (Deng et al., 2024). While continuous cropping is a common agricultural practice aimed at enhancing land-use efficiency, its long-term application often manifests as “continuous cropping obstacles (CCOs)”, characterized by yield decline, quality degradation, and increased susceptibility to pests and diseases (Wang et al., 2024; Wacal et al., 2019). Nevertheless, the specific impact of continuous cropping on lavender growth remains poorly understood. Consequently, systematically elucidating the continuous cropping effects in lavender is crucial for optimizing cultivation practices and ensuring the sustainable development of this industry.

CCOs arising from long-term monoculture, represent a systemic ecological problem (Ma et al., 2025; Zeeshan Ul Haq et al., 2023). Their detrimental effects on crop yield and quality have been substantiated across various crop species (Liu et al., 2025a; Wacal et al., 2019), although the intensity and manifestation of these effects vary depending on the specific biological characteristics of the crop (Wang et al., 2024). At the yield level, CCOs mainly reduce biomass and economic yield by increasing soil-borne diseases and pests and altering soil quality (Arafat et al., 2019). A recent meta-analysis indicates that CCOs cause an average crop yield reduction of 22% (Wang et al., 2024). For instance, even under adequate fertilization regimes, soybean yields declined by approximately 10–40% after three years of continuous cropping (Yang et al., 2024a). Similarly, continuous cropping significantly inhibited potato seedling and adventitious root growth, resulting in a pronounced yield decrease of 24.43% (Ma et al., 2025). Notably, recent research reveals that not all continuous cropping systems inevitably lead to CCOs (Wang et al., 2024). In specific instances, continuous cropping can foster the development of disease-suppressive soils (Jing et al., 2022), subsequently enhancing yield (Wang et al., 2024). Critically, regardless of whether the impact of continuous cropping on crop yield and quality is ultimately positive or negative, these effects are mediated through alterations in soil physicochemical properties.

Soil pH, a critical physicochemical indicator, significantly affects nutrient availability and microbial activity (Wang and Kuzyakov, 2024). Continuous cropping often causes soil acidification, impairing soil buffering capacity (Arafat et al., 2019), mainly due to root exudate accumulation (e.g., phenolic acids) (Yang et al., 2024b), and long-term chemical fertilizer application (Juo et al., 1995). Nutrient imbalance is another key factor of CCOs (Gan et al., 2025). Long-term monoculture leads to selective nutrient uptake, disrupting ecological stoichiometric ratios (e.g., C/N, C/P) and interfering with plant metabolism (Gan et al., 2025), such as phosphorus depletion in continuous alfalfa cropping (Yang et al., 2023). Additionally, continuous accumulation of phytotoxic allelochemicals from roots induces autotoxicity (Gan et al., 2025), inhibiting plant growth and driving CCOs in multiple crops (Gan et al., 2025; Ma et al., 2025; Xuan et al., 2024). These factors collectively degrade soil quality and cause CCOs. Soil enzyme activity, a vital “biological indicator” of soil functionality, mediates continuous cropping’s impact on crop yield and quality (JaiPaul et al., 2014), reflecting soil fertility and ecological integrity by facilitating organic matter decomposition and nutrient transformation (Daunoras et al., 2024). Its impairment results from altered soil physicochemical properties and microbial community structure (Zeeshan Ul Haq et al., 2025), with key enzyme activities declining under continuous cropping (Gan et al., 2025; Wacal et al., 2019). Short-term peanut monoculture reduces LAP activity, impairing nitrogen mineralization and yield (Yu et al., 2024). Quantifying this inhibition is thus crucial for assessing soil health and elucidating CCO mechanisms (Ahsan et al., 2024). Moreover, soil microbial community structure and function responses to continuous cropping are fundamental to CCO formation (Chen et al., 2025).

Soil microbial communities regulate crop growth and quality via nutrient transformation, phytohormone regulation, disease suppression, and metabolic regulation (Fang et al., 2025; Nettles et al., 2016). Continuous cropping reshapes microbial community structure through environmental filtering and interspecies interactions (Han et al., 2024; Xuan et al., 2024). On one hand, monoculture-induced soil physicochemical changes (e.g., pH shifts, nutrient imbalance) select for dominant, adaptable microbial groups (Ma et al., 2025). On the other hand, the allelopathic effects of root exudates can alter the complexity and stability of microbial co-occurrence networks (Kudjordjie et al., 2019). Existing studies confirm continuous cropping reduces soil bacterial diversity, simplifies co-occurrence networks, and diminishes soil functional redundancy (Cao et al., 2025; Yu et al., 2024). Deciphering microbe-plant interactions is key to understanding continuous cropping effects on yield and quality (Xi et al., 2025; Xuan et al., 2024). Microbial nutrient limitations can alter microbial metabolic strategies, thereby regulating soil nutrient supply and plant productivity (Čapek et al., 2018; Soong et al., 2020), with vegetation productivity more strongly correlated with microbial phosphorus limitation than climatic or soil variables globally (Fay et al., 2025). Additionally, microbial co-occurrence network complexity is linked to soil functional stability, with more complex networks enhancing disturbance resistance and functional redundancy (Wang et al., 2023; Kajihara and Hynson, 2024). Microbial community dysbiosis impairs nutrient transformation efficiency (Fang et al., 2025) and modifies the rhizosphere microenvironment, making the microbiome a pivotal hub regulating continuous cropping effects (Xuan et al., 2024).

Despite significant progress in understanding general continuous cropping effects (Cao et al., 2025; Ma et al., 2025; Xuan et al., 2024), systematic knowledge regarding lavender remains elusive. In recent years, driven by sustained market demand, lavender cultivation areas in China, particularly in the Yili River Valley of Xinjiang—China’s largest production base—have expanded considerably. Consequently, this study focuses on the primary lavender-producing areas within the Xinjiang Yili River Valley. Three representative regions were selected for investigation. Experimental treatments comprised monoculture duration gradients of 1 year, 3 years, and 6 years. We propose the following hypotheses: (1) Long-term continuous cropping of lavender will lead to a decline in both the quality characteristics and yield of essential oil. (2) Long-term continuous cropping will degrade soil nutrient levels and enzyme activity. (3) Long-term continuous cropping will reduce the diversity of the soil bacterial community as well as the complexity of its co-occurrence network.

## 2 Materials and methods

### 2.1 Study area description

This study was conducted in three major lavender cultivation zones within the Ili Kazakh Autonomous Prefecture, Xinjiang, China (**Supplementary Figure 1**): (1) Yining Agricultural Research Institute Experimental Site (YL) (43°55’N, 81°04’E): Characterized by a temperate continental climate, featuring abundant solar and thermal resources, long sunshine duration, substantial accumulated temperature, and significant diurnal temperature variation. The mean annual temperature (MAT) is 8.2 °C. The frost-free period spans 150–160 days. Measured baseline soil properties are as follows: pH = 7.83; Soil organic carbon (SOC) = 7.29 g kg^−^¹; Total nitrogen (TN) = 1.34 g kg^−^¹; Total phosphorus (TP) = 1.50 g kg^−^¹. (2) Tianshan Flower Sea, Kashi Town, Yining County (YN) (43°45’N, 81°20’E): Also experiencing a temperate continental climate, notable for its rich heat resources and sufficient sunlight. The long-term MAT is 8.1 °C, with an average frost-free period of 159 days. Soil baseline properties: pH = 7.57; SOC = 5.78 g kg^−^¹; TN = 1.23 g kg^−^¹; TP = 1.62 g kg^−^¹. (3) Sigong Village, Lucaogou Town, Huocheng County (HC) (44°05’N, 80°43’E): Located at 80.89°E, 44.05°N, within a temperate continental climate zone. The long-term MAT is 8.8 °C, with an average frost-free period of 165 days. Soil baseline properties: pH = 7.83; SOC = 8.15 g kg^−^¹; TN = 1.21 g kg^−^¹; TP = 1.49 g kg^−^¹. More details can be found in **Supplementary Table 3**. According to FAO soil classification, the soil types in the three sampling areas are all grey calcareous.

### 2.2 Experimental design and sample collection

The test lavender cultivar was ‘French Blue’, the predominant local variety. Experimental plots with monoculture durations of 1 year (Y1), 3 years (Y3), and 6 years (Y6) were selected for investigation. A factorial design was employed: 3 sites × 3 monoculture durations × 3 replicates = 27 experimental plots. Each plot measured 100 m² (10 m × 10 m). Consistent agronomic practices (irrigation, fertilization, pest and disease control) were applied uniformly across all plots. This standardization ensured the homogenization of environmental variables other than the target factor of monoculture duration.

Plant Sample Collection: Plant samples were collected during the full bloom stage (June 15th, 2024). Within each plot, three randomly selected 1 m × 1 m quadrats were established. Whole fresh lavender plants were cut at 10 cm below the spike (Verma et al., 2010). After collection, the plant samples were placed neatly into sample bags, stored temporarily in a refrigerator at 4 °C, and the determination of biomass yield per unit area (kg ha^−^¹) and extraction of essential oil were completed within 48 hours.

Soil Sample Collection (June 15th, 2024): Soil sampling followed a randomized protocol within each plot. Three random sampling points were selected per plot. Soil cores were extracted using a 5-cm diameter soil auger to the required depths (0–20 cm and 20–40 cm). After collection, visible plant residues, stones, and other debris were manually removed from the soil cores. The soil from the three points per plot was then composited, thoroughly homogenized, and passed through a 2-mm sieve to form one representative sample per plot. This composite sample was subsequently subdivided into two aliquots. One aliquot was air-dried at room temperature for the analysis of soil physicochemical properties. The other aliquot was immediately stored at -20 °C for subsequent determination of soil enzyme activities and soil microbial community analysis (e.g., DNA extraction and sequencing).

### 2.3 Measured parameters and methods

#### 2.3.1 Analysis of soil physicochemical properties

Soil pH was measured using a DELTA 320 pH meter (Mettler-Toledo, Switzerland) in a soil suspension prepared with deionized water at a soil-to-water ratio of 1:2.5 (w/v). Soil organic carbon (SOC) content was determined by the potassium dichromate (K_2_Cr_2_O_7_)-concentrated sulfuric acid (H_2_SO_4_) volumetric method with external heating (Zeng et al., 2025). For total nitrogen (TN) and total phosphorus (TP) analysis, air-dried soil samples were finely ground and sieved; TN content was quantified using an automatic Kjeldahl nitrogen determination apparatus following wet digestion with concentrated H_2_SO_4_ (Zeng et al., 2024a), while TP content was measured via the molybdenum-antimony colorimetric method after wet digestion with a concentrated H_2_SO_4_-perchloric acid (HClO_4_) mixture (Zeng et al., 2024a).

#### 2.3.2 Determination of soil enzyme activity and calculation of nutrient limitation

The activities of six hydrolytic enzymes—comprising carbon (C)-acquisition enzymes (β-1,4-glucosidase, BG; cellobiohydrolase, CBH; β-xylosidase, BX), nitrogen (N)-acquisition enzymes (β-1,4-N-acetylglucosaminidase, NAG; leucine aminopeptidase, LAP), and the organic phosphorus (P)-acquisition enzyme (alkaline phosphatase, AP)—were determined using a multifunctional microplate reader. Assays were conducted in 96-well black microplates with fluorescence measured at excitation and emission wavelengths of 365 nm and 450 nm, respectively. Enzyme activities are expressed as nanomoles of substrate hydrolyzed per gram of dry soil per hour (nmol·g^−^¹·h^−^¹). Detailed protocols are provided in Text S1.

Microbial nutrient limitations were modeled using enzymatic vector analysis by plotting the ratios of C-acquiring to N-acquiring enzymes (C:Nenzyme) versus C-acquiring to P-acquiring enzymes (C:Penzyme) (Moorhead et al., 2016). Resource limitations were quantitatively assessed by calculating vector length (indicating C limitation) and vector angle (reflecting P limitation when >45° and N limitation when<45°), with the specific computational formulas detailed as follows (Equation 1):

(1) $$\text{Vector length}=\sqrt{{\left(\frac{\text{BG}+\text{CBH}+\text{BX}}{\text{BG}+\text{CBH}+\text{BX}+\text{AP}}\right)}^{2}+{\left(\frac{\text{BG}+\text{CBH}+\text{BX}}{\text{BG}+\text{CBH}+\text{BX}+\text{NAG}+\text{LAP}}\right)}^{2}}$$

Vector angle (Equation 2)

(2) $$=\text{Degress}\left[\text{ATAN}2\left(\frac{\text{BG}+\text{CBH}+\text{BX}}{\text{BG}+\text{CBH}+\text{BX}+\text{AP}},\frac{\text{BG}+\text{CBH}+\text{BX}}{\text{BG}+\text{CBH}+\text{BX}+\text{NAG}+\text{LAP}}\right)\right]$$

#### 2.3.3 Soil bacterial sequencing

Genomic DNA was extracted from soil samples using the TIANamp Soil DNA Kit (Tiangen Biotech, China), with quality-verified extracts stored at –20 °C. The bacterial 16S rRNA gene was amplified via PCR with universal primers 519F (5′-CAGCMGCCGCGGTAATWC-3′) and 907R (5′-CCGTCAATTCMTTTRAGTTT-3′). PCR products were purified, validated by 2% agarose gel electrophoresis, and target bands were excised for recovery. Purified amplicons from all samples were pooled in equimolar concentrations. Sequencing libraries were prepared using the TruSeq Nano DNA LT Library Prep Kit (Illumina, USA), with uniquely indexed libraries diluted to appropriate concentrations, pooled proportionally based on sequencing depth requirements, denatured into single-stranded DNA with NaOH, and sequenced on an Illumina MiSeq platform (Personal Biotechnology Co., Ltd., Shanghai) for paired-end 2×300 bp reads. Raw sequences were processed by merging paired-end reads, quality filtering, and chimera removal. High-quality sequences were clustered into operational taxonomic units (OTUs) at a 97% similarity threshold using UPARSE. Taxonomic classification was assigned by aligning representative OTU sequences against the SILVA 138 database (or Greengenes), yielding community composition profiles across kingdom, phylum, class, order, family, genus, and species levels.

#### 2.3.4 Essential oil extraction and compositional analysis

Lavender essential oil was extracted via hydrodistillation using a Clevenger-type apparatus (Liu et al., 2025b). Fresh flowers were mixed with deionized water at a flower-to-water ratio of 1:12 (w/v). The mixture was transferred to a 2000 mL round-bottom flask and heated under constant-temperature conditions until boiling commenced. Distillation was maintained for 90 minutes after boiling initiation. The crude oil (upper layer) collected in the condenser arm was separated using a Dean-Stark trap, subsequently dehydrated with anhydrous sodium sulfate (Na_2_SO_4_) to remove residual water, and stored in light-protected vials at 4 °C. This extraction procedure was performed in five biological replicates per experimental plot. The essential oil yield rate was calculated as follows (Equation 3):

(3) $$\text{Oil yield rate }(\text{mL/kg})=\frac{\text{Oil extraction volume }(\text{mL})}{\text{Flower weight }(\text{kg})}$$

GC-MS Analysis of Essential Oil Components: The primary constituents of lavender essential oil—linalool, camphor, linalyl acetate, and lavandulyl acetate—were analyzed using gas chromatography-mass spectrometry (GC-MS; Agilent 7890B GC/5977B MSD) with an HP-5MS capillary column (30 m × 0.25 mm × 0.25 μm). Chromatographic conditions included: ultra-high-purity helium (99.999%) carrier gas at 1.0 mL/min constant flow; split injection (10:1 ratio) at 250 °C with 1 μL injection volume; and a temperature program initiating at 60 °C (hold 2 min), ramping at 5 °C/min to 180 °C (hold 5 min), then at 10 °C/min to 250 °C (hold 10 min). Mass spectrometric detection employed electron ionization (EI) at 70 eV with ion source temperature 230 °C, quadrupole temperature 150 °C, transfer line temperature 280 °C, scan range m/z35–500, and 3 min solvent delay (Cheng et al., 2025).

Compound Identification and Quantitative Validation: Compound identification was achieved by matching mass spectra against both the NIST 2020 mass spectral library and a curated lavender-specific compound database using Agilent MassHunter software, with retention time confirmation against authentic standards. Relative abundances were calculated via normalized peak area quantification. Method validation confirmed linear responses for all target analytes (*R*² ≥ 0.995) and chromatographic resolution ≥1.5 between critical peak pairs, ensuring quantitative accuracy. Each sample underwent triplicate technical injections, with mean values reported for final compositional data.

### 2.4 Statistical analysis

Raw data were organized using Microsoft Excel 2023, with all statistical analyses performed in R (version 4.4.2). Prior to significance testing, data normality was assessed via the Shapiro-Wilk test, and homogeneity of variances was evaluated using Levene’s F-test; variables violating assumptions underwent logarithmic transformation when necessary. Differences in soil physicochemical properties, biological attributes, essential oil yield, and quality across monoculture durations and sites were determined by two-way analysis of variance (Two-way ANOVA) followed by Duncan’s multiple range test for *post-hoc* comparisons. To quantify the independent effects of continuous cropping, a linear mixed-effects model was constructed with the experimental site as the random effect and the years as the fixed effect (**Supplementary Figures 2-5**). We used the R packages ggplot2, vegan, and ggalluvialto generate bar plots, non-metric multidimensional scaling (NMDS) ordinations, and community composition-stacked bar plots for data visualization. Microbial co-occurrence network topology—including node count, edge number, clustering coefficient, average degree, network density, average path length, centrality, and modularity—was calculated using the microecoand igraphpackages, with network visualizations rendered in igraphincorporating node coloring and edge attributes (Yuan et al., 2021). Microbial co-occurrence networks were constructed using Spearman correlation analysis, with a correlation threshold of |r| > 0.7 and a significance level of P< 0.01 set as the criteria for significant correlation to ensure the reliability of the networks (Liu et al., 2023b). The complexity of the co-occurrence networks was characterized by principal component analysis (PCA1) of the calculated topological features of the co-occurrence networks. In addition, sampling, network reconstruction, and conservation rate calculation were completed using the microeco and igraph packages in R, which are compatible with the existing network analysis workflow. Multiple samplings were performed to reduce random errors and ensure the reliability of the network structure and topological features. Random forest analysis (with 500 trees and mtry = 5) and Pearson correlation analysis were conducted using the “randomForest” and “psych” packages in R to evaluate the effects of soil physicochemical properties, enzyme activities, nutrient limitations, microbial diversity, and network complexity on essential oil yield and its main components. Based on the results of correlation and random forest analyses, the core driving factors closely related to essential oil yield were screened out. The “plspm” package in R was used to construct a partial least squares path model (PLS-PM) to clarify the direct and indirect action paths between latent variables (soil physicochemical properties, soil enzyme activities, microbial nutrient limitations, network topological features, and essential oil yield).

## 3 Results

### 3.1 Effects of continuous cropping duration on lavender essential oil yield and composition

**Figure 1:**
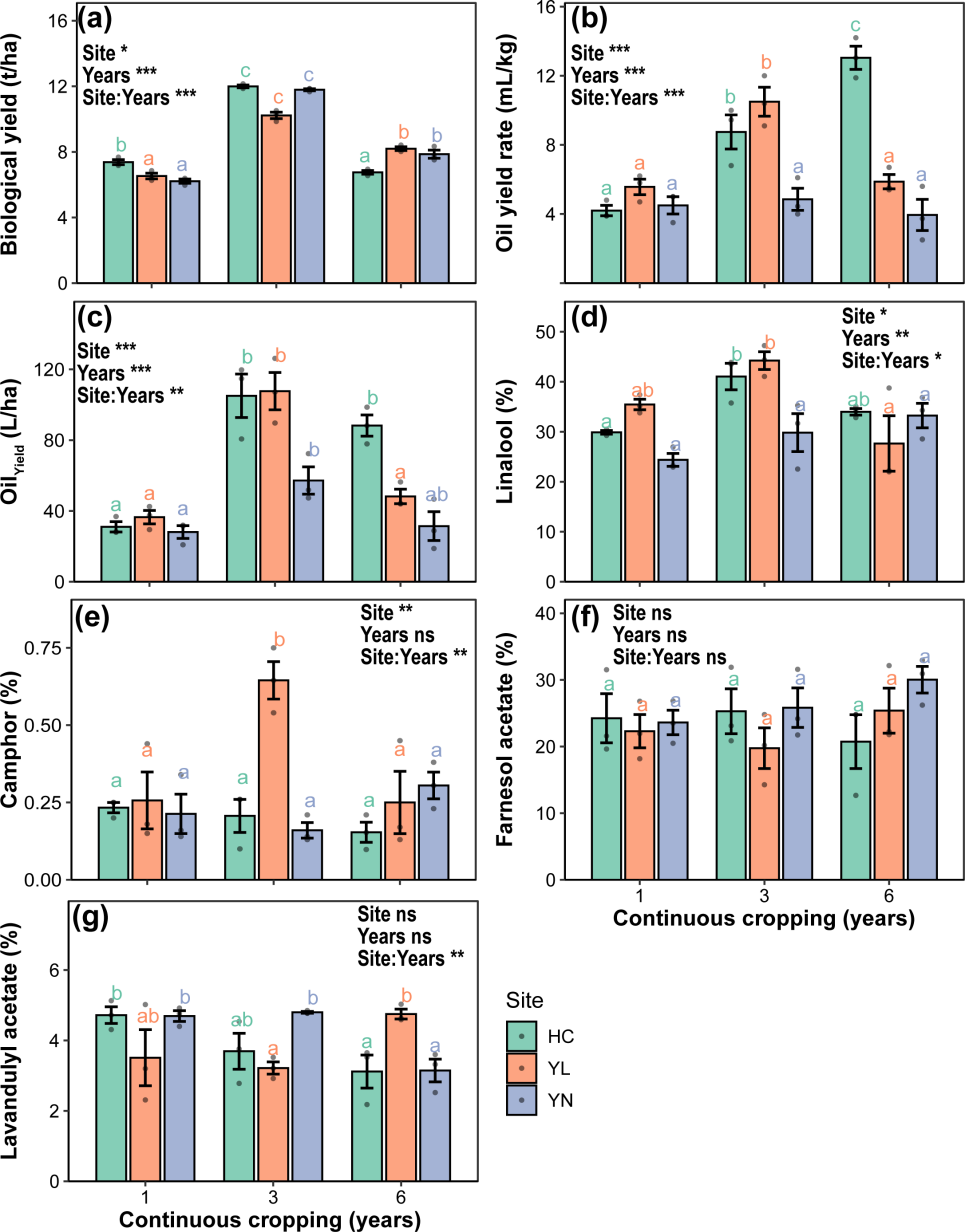
Effects of continuous cropping duration on lavender biomass yield and essential oil composition. Bars colored distinctly represent different sampling sites. Data are presented as mean ± standard deviation (SD). Different superscript letters indicate significant differences among continuous cropping durations within a given site (P < 0.05). Results of two-way ANOVA demonstrate the effects of Sampling Site (Site), Continuous Cropping Duration (Years), and their Interaction (Site × Years). ns indicates P > 0.05, not significantly different. *, **, *** represent significant differences at P < 0.05, < 0.01, < 0.001 levels, respectively. **(a)** Biological yield: Dry weight biomass yield of lavender; **(b)** Oil yield rate: The amount of essential oil produced per kilogram of lavender; **(c)** Oil_Yield_: Essential oil yield per unit area; **(d-g)**: Changes in the content of key essential oil components.

Two-way ANOVA revealed significant differential responses of essential oil yield metrics to both cultivation site and monoculture duration (Years) (**Figure 1**). For yield parameters: Oil yield rate exhibited significant main effects of Site (F = 31.05, *P<* 0.001) and Years (F = 21.18, *P<* 0.001), with a significant interaction (*P<* 0.001) (**Figure 1b**). In HC, the 6-year monoculture treatment (13.04 ± 0.67 mL/kg) significantly exceeded both 3-year and 1-year durations (*P<* 0.05). YL showed peak extraction at 3 years (10.50 ± 0.67 mL/kg, *P<* 0.05 vs. other durations), while YN exhibited no significant temporal differences (*P>* 0.05). Biomass yield (Yield) was profoundly influenced by Years (F = 792.43, *P<* 0.001) (**Figure 1a**), consistently following the order 3-year > 6-year > 1-year across all sites. Total essential oil yield (Oil_Yield_) was strongly driven by Years (F = 48.34, *P<* 0.001), peaking at the 3-year duration in HC and YL (**Figure 1c**). In YN, the 3-year yield (57.19 ± 7.27 L/ha) significantly surpassed the 1-year treatment (*P<* 0.05).

Linalool content demonstrated significant main effects of Site (F = 5.47, *P<* 0.05) and Years (F = 8.35, *P<* 0.01), plus a significant interaction (F = 4.21, *P<* 0.05) (**Figure 1d**). Peak linalool concentrations occurred at the 3-year duration in HC (41.04 ± 2.68%) and YL (44.23 ± 2.68%), both significantly higher than 1-year values (*P<* 0.05), while YN showed no temporal variation (*P>* 0.05). Camphor was affected by Site (F = 8.22, *P<* 0.01) and Site × Years interaction (F = 6.51, *P<* 0.01), with only YL exhibiting a significant 3-year increase (0.65 ± 0.06%, *P<* 0.05) (**Figure 1e**). Farnesol acetate showed no significant differences across durations (range: 19.75–30.04%) (**Figure 1f**). Lavandulyl acetate displayed a significant Site × Years interaction (F = 6.22, *P<* 0.01), decreasing with monoculture duration in HC and YN (**Figure 1g**). Critically, continuous cropping enhanced oil extraction rates without altering the primary chemical composition of essential oils.

### 3.2 Impact of monoculture on soil nutrients

**Figure 2:**
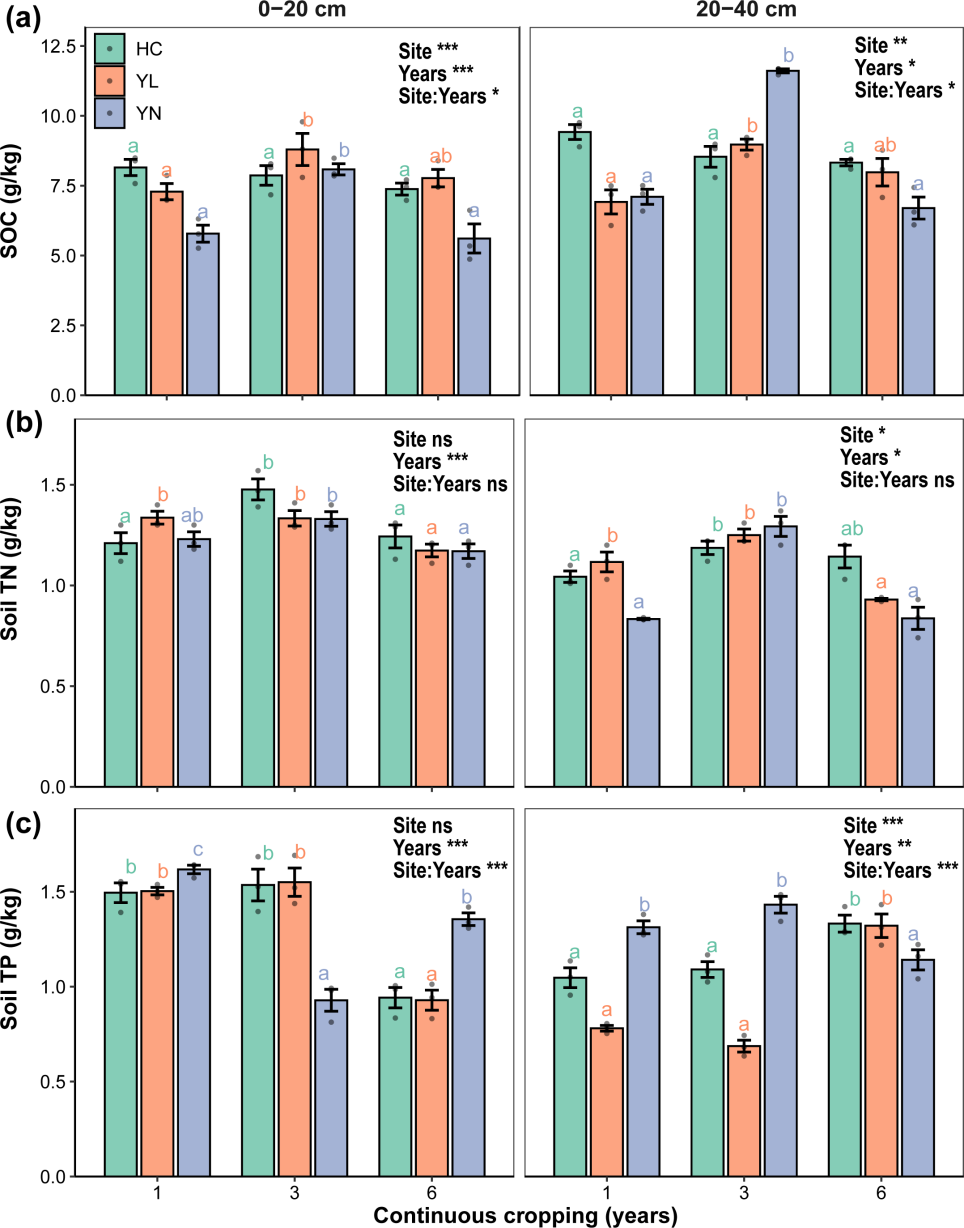
Effects of continuous cropping duration on soil nutrient. Bars colored distinctly represent different sampling sites. Data are presented as mean ± standard deviation (SD). Different superscript letters indicate significant differences among continuous cropping durations within a given site (P < 0.05). Results of two-way ANOVA demonstrate the effects of Sampling Site (Site), Continuous Cropping Duration (Years), and their Interaction (Site × Years). ns indicates P > 0.05, not significantly different. *, **, *** represent significant differences at P < 0.05, < 0.01, < 0.001 levels, respectively. **(a)** SOC: Soil Organic Carbon; **(b)** TN: Total Nitrogen; **(c)** TP: Total Phosphorus.

Two-way ANOVA demonstrated significant effects of monoculture duration, site, and their interaction on soil nutrients across both 0–20 cm and 20–40 cm layers (*P<* 0.05), with regional and temporal effects varying by parameter and depth (**Figure 2**). In the 0–20 cm layer, SOC was predominantly influenced by site (F = 14.84, *P<* 0.001), showing no temporal variation in Huocheng (HC) but peaking at 3 years in Yining County (YL: 8.80 ± 0.57 g/kg) and Yining City (YN: 8.08 ± 0.20 g/kg; *P<* 0.05 vs. other durations) (**Figure 2a**). TN was strongly driven by duration (F = 14.77, *P<* 0.001), peaking at 3 years across all sites, with YL and YN significantly exceeding 6-year levels (*P<* 0.05) (**Figure 2b**). TP exhibited significant duration × site interaction (F = 55.50, *P<* 0.001), where HC and YL showed higher TP at 1–3 years (1.49–1.55 g/kg) than at 6 years, while YN displayed a high–low–high trajectory with recovery to 1.36 ± 0.03 g/kg at 6 years (**Figure 2c**). In the 20–40 cm layer, SOC and TN were primarily controlled by duration (F = 37.72 and 43.71, respectively; *P<* 0.001), both peaking at 3 years in YN (11.61 ± 0.32 g/kg and 1.29 ± 0.04 g/kg) (**Figures 2a, b**); TP was dominated by interaction effects, with higher values at 6 years in HC/YL but peaking at 3 years in YN (1.43 ± 0.04 g/kg; *P<* 0.05) (**Figure 2c**). C/P ratios in both layers were significantly affected by duration and interaction (*P<* 0.01, **Supplementary Table 1**), with 6-year durations generally elevating ratios versus 3-year durations (*P<* 0.05). Surface pH (0–20 cm) varied regionally (F = 4.72, P = 0.023) but not temporally (7.57–8.17), while subsurface pH (20–40 cm) remained stable (7.87–8.17) across all factors (*P>* 0.05, **Supplementary Table 1**).

### 3.3 The impact of continuous cropping on soil enzyme activity and microbial nutrient limitation

**Figure 3:**
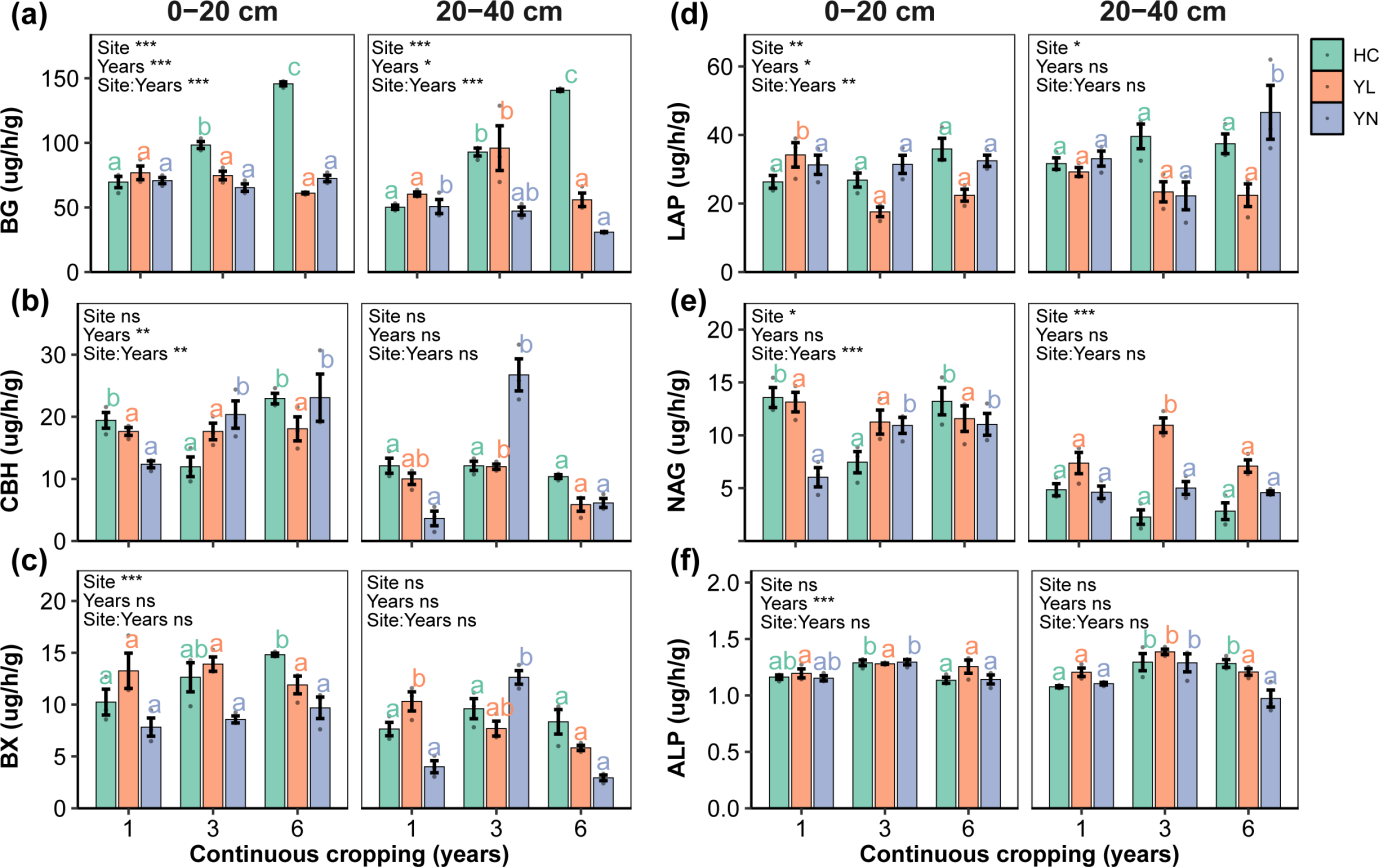
Effects of continuous cropping duration on soil enzyme activity. Bars colored distinctly represent different sampling sites. Data are presented as mean ± standard deviation (SD). Different superscript letters indicate significant differences among continuous cropping durations within a given site (P < 0.05). Results of two-way ANOVA demonstrate the effects of Sampling Site (Site), Continuous Cropping Duration (Years), and their Interaction (Site × Years). ns indicates P > 0.05, not significantly different. *, **, *** represent significant differences at P < 0.05, < 0.01, < 0.001 levels, respectively. **(a)** BG: β-1,4-glucosidase, **(b)** CBH: Cellobiohydrolase, **(c)** BX: β-xylosidase, **(d)** LAP: Leucine aminopeptidase, **(e)** NAG: β-1,4-N-acetylglucosaminidase, **(f)** ALP: alkaline phosphatase.

Two-way ANOVA revealed that β-glucosidase (BG) activity in the 0–20 cm layer was significantly influenced by monoculture duration, region, and their interaction (*P<* 0.001) (**Figure 3a**). HC, BG activity increased progressively with duration, peaking at 6 years (145.70 ± 3.12 nmol g^−^¹ h^−^¹), significantly exceeding 3-year (98.34 ± 3.12) and 1-year (69.69 ± 3.12) levels (*P<* 0.05), while other regions showed no significant temporal trends. Similar duration-dependent patterns were observed for BG in the 20–40 cm layer. CBH activity in the 0–20 cm layer was strongly driven by duration (F = 56.19, *P<* 0.001), with YN exhibiting significant increases over time, peaking at 3 years (11.96 ± 1.20 nmol g^−^¹ h^−^¹) (**Figure 3b**). Conversely, BX and NAG activities showed no response to duration (*P>* 0.05) (**Figures 3c, e**). ALP was significantly affected by duration (F = 12.61, *P<* 0.001), with HC and YN showing peak activity at 3 years (1.29 ± 0.03 nmol g^−^¹ h^−^¹), significantly higher than 1- and 6-year treatments (*P<* 0.05) (**Figure 3d**). Collectively, enzymatic responses to continuous cropping exhibited region-specific patterns, yet no significant inhibitory effects were observed across all regions (**Figure 3**).

**Figure 4:**
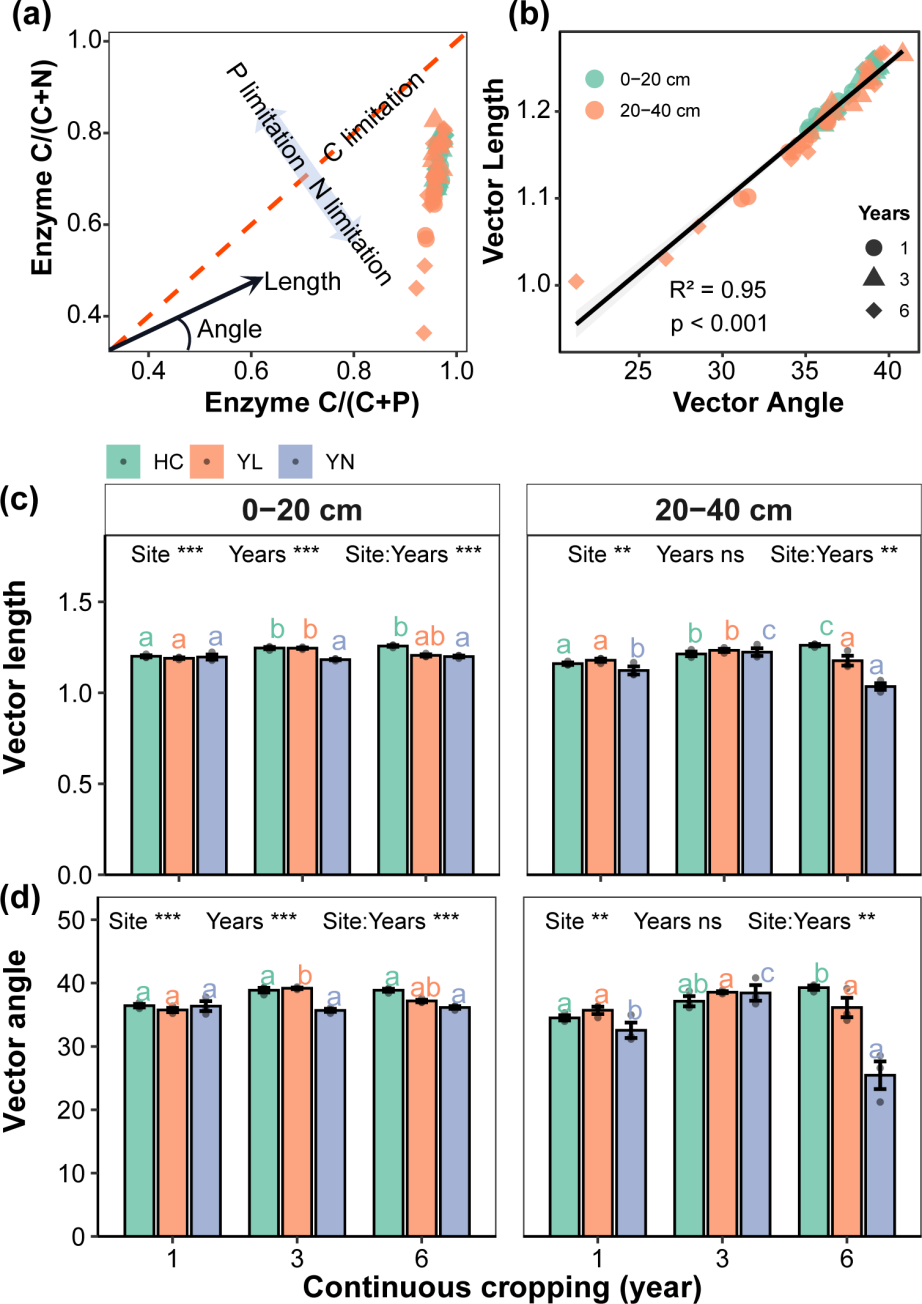
Enzyme vector analysis modeling of microbial resource limitations **(a)** and soil enzyme vector angle/length characteristics **(b-d)** under continuous cropping. Bars colored distinctly represent different sampling sites. Data are presented as mean ± standard deviation (SD). Different superscript letters indicate significant differences among continuous cropping durations within a given site (*P* < 0.05). Results of two-way ANOVA demonstrate the effects of Sampling Site (Site), Continuous Cropping Duration (Years), and their Interaction (Site × Years). ns indicates *P* > 0.05, not significantly different. *, **, *** represent significant differences at *P* < 0.05,< 0.01,< 0.001 levels, respectively. *R* represents goodness-of-fit, and *P* indicates significance level. The gray areas represent 95% confidence intervals.

Enzymatic stoichiometry revealed pronounced N limitation across the study region (**Figure 4a**). Two-way ANOVA demonstrated that both vector angle and vector length were significantly influenced by monoculture duration, region, and their interaction (*P<* 0.01) (**Figures 4c, d**). In the 0–20 cm layer, HC exhibited significantly higher vector angles at 3 and 6 years (38.87–38.89°) versus 1 year (36.43°; *P<* 0.05), while YL showed a gradient of 1 year (35.75°)< 6 years (37.17°)< 3 years (39.18°) (*P<* 0.05), with no temporal differences in YN. Vector length peaked at 3 years in HC (1.25) and YL (1.24), significantly exceeding 1-year values (1.19–1.20; *P<* 0.05), while YN remained stable. In the 20–40 cm layer, HC displayed higher vector angles at 6 years (39.27°) than at 1 year (34.51°; *P<* 0.05), whereas YN followed 6 years (25.45°)< 1 year (32.55°)< 3 years (38.45°) (*P<* 0.05). Collectively, long-term monoculture increased vector angles and lengths, intensifying microbial C limitation while alleviating N limitation. Linear regression confirmed a significant positive correlation between vector angle and length (*P<* 0.001) (**Figure 4b**).

### 3.4 The impact of monoculture on soil bacterial communities

**Figure 5:**
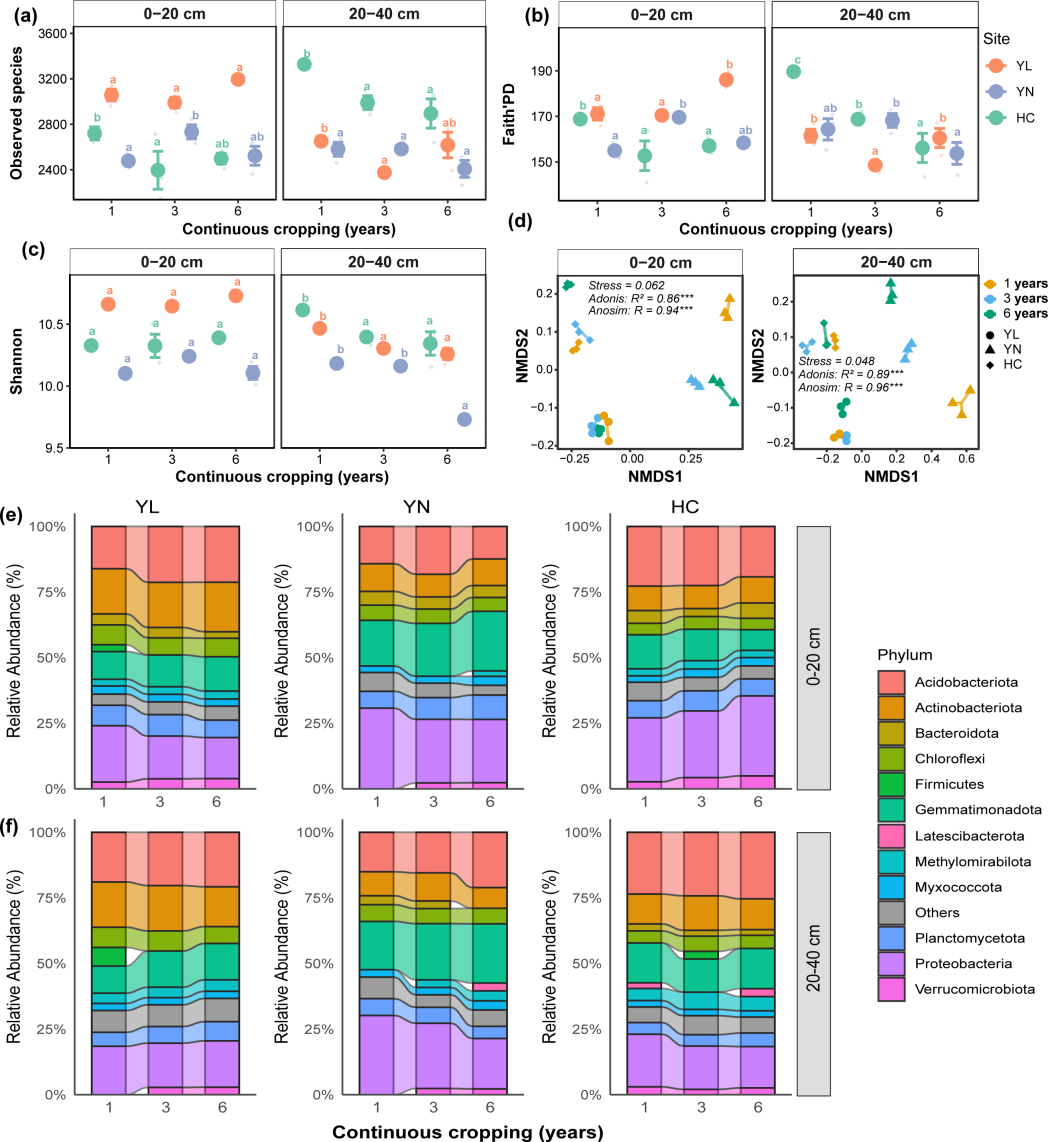
Bacterial diversity indices **(a-d)** and phylum-level relative abundance profiles **(e, f)** under continuous cropping. **(a-c)** Points with distinct colors denote different sampling sites. Data are presented as mean ± standard deviation (SD). Different superscript letters indicate statistically significant differences among continuous cropping durations within a given site (*P* < 0.05). **(d)** Non-metric Multidimensional Scaling (NMDS) ordination plot displays dissimilarities in bacterial community composition (β-diversity) among treatments. Significance was tested using Adonis and Anosim analyses (*P* < 0.001). **(e, f)** Stacked bar plots illustrate shifts in relative abundance at the bacterial phylum level, with distinct colors representing different phyla.

Two-way ANOVA revealed region-dependent responses of soil microbial diversity to monoculture duration (**Figures 5a-c**). In the 0–20 cm layer, phylogenetic diversity (Faith’PD) was significantly influenced by region (F = 33.17, *P<* 0.001) and site × duration interaction (F = 13.41, *P<* 0.001): HC peaked at 1 year (*P<* 0.05 vs. 3–6 years), YL maximized at 6 years, and YN peaked at 3 years (*P<* 0.05). Observed species richness decreased significantly only in HC from 1 to 3 years (*P<* 0.05), while the Shannon index showed no temporal differences. In the 20–40 cm layer, all three diversity metrics declined significantly with increasing duration (*P<* 0.01), indicating greater sensitivity in subsurface soils. Regional environments modulated duration effects through significant interactions.

β-diversity shifted markedly under continuous cropping (**Figure 5d**). NMDS ordination (stress = 0.0615–0.048) demonstrated significant compositional divergence by duration (*P<* 0.001, Adonis/Anosim). Communities clustered regionally on NMDS axes but exhibited directional succession along duration gradients. At the phylum level, *Proteobacteria*, *Actinobacteriota*, and *Acidobacteriota* dominated across all groups (**Figures 5e, f**). Notably, *Proteobacteria* declined with duration in both soil layers, while *Verrucomicrobiota* accumulated over time. These shifts confirm that continuous cropping restructures bacterial phylum-level composition, though most changes were mediated by region-dependent effects.

**Figure 6:**
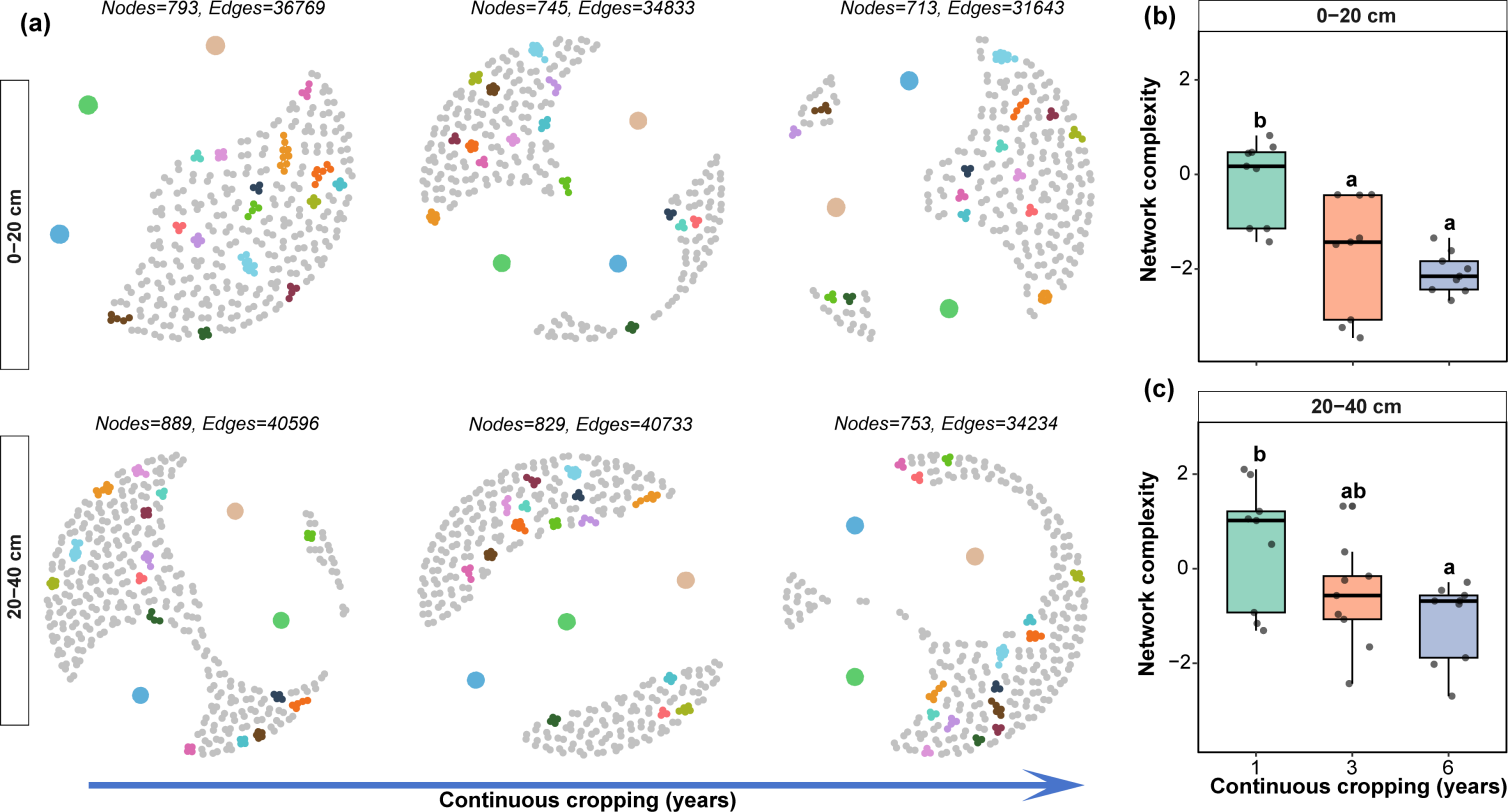
Effects of continuous cropping duration on bacterial co-occurrence network topology **(a)** and complexity indices **(b, c)**. **(a)** Large network modules (≥5 nodes) are displayed in distinct colors, with smaller modules shown in gray. Detailed topological properties are provided in **Supplementary Table 2**. **(b, c)** Box plots illustrate shifts in network complexity indices across cropping durations. Different superscript letters indicate statistically significant differences among continuous cropping durations (*P* < 0.05).

Analysis of co-occurrence networks revealed systematic changes in bacterial association structures with increasing monoculture duration (**Figure 6**). Across both soil layers (0–20 cm and 20–40 cm), prolonged continuous cropping reduced network complexity, evidenced by declining node counts and edge numbers—indicating simplified microbial interactions (**Figure 6a**). Principal Component Analysis (PCA1) of eight topological metrics (node count, edge number, clustering coefficient, average degree, network density, average path length, centrality, and modularity) quantitatively confirmed that extended monoculture significantly reduced microbial network complexity in both soil layers (*P<* 0.05), reflecting fundamental restructuring of microbial community associations under long-term cultivation (**Figure 6b**). We performed functional annotation of bacteria using FAPROTAX and found that continuous cropping increased bacterial functions such as nitrification, sulfate respiration, and sulfur oxidation in the 0–20 cm soil layer, but decreased these bacterial functions in the 20–40 cm soil layer (**Supplementary Figure 6**).

### 3.5 Drivers of lavender essential oil yield and quality

**Figure 7:**
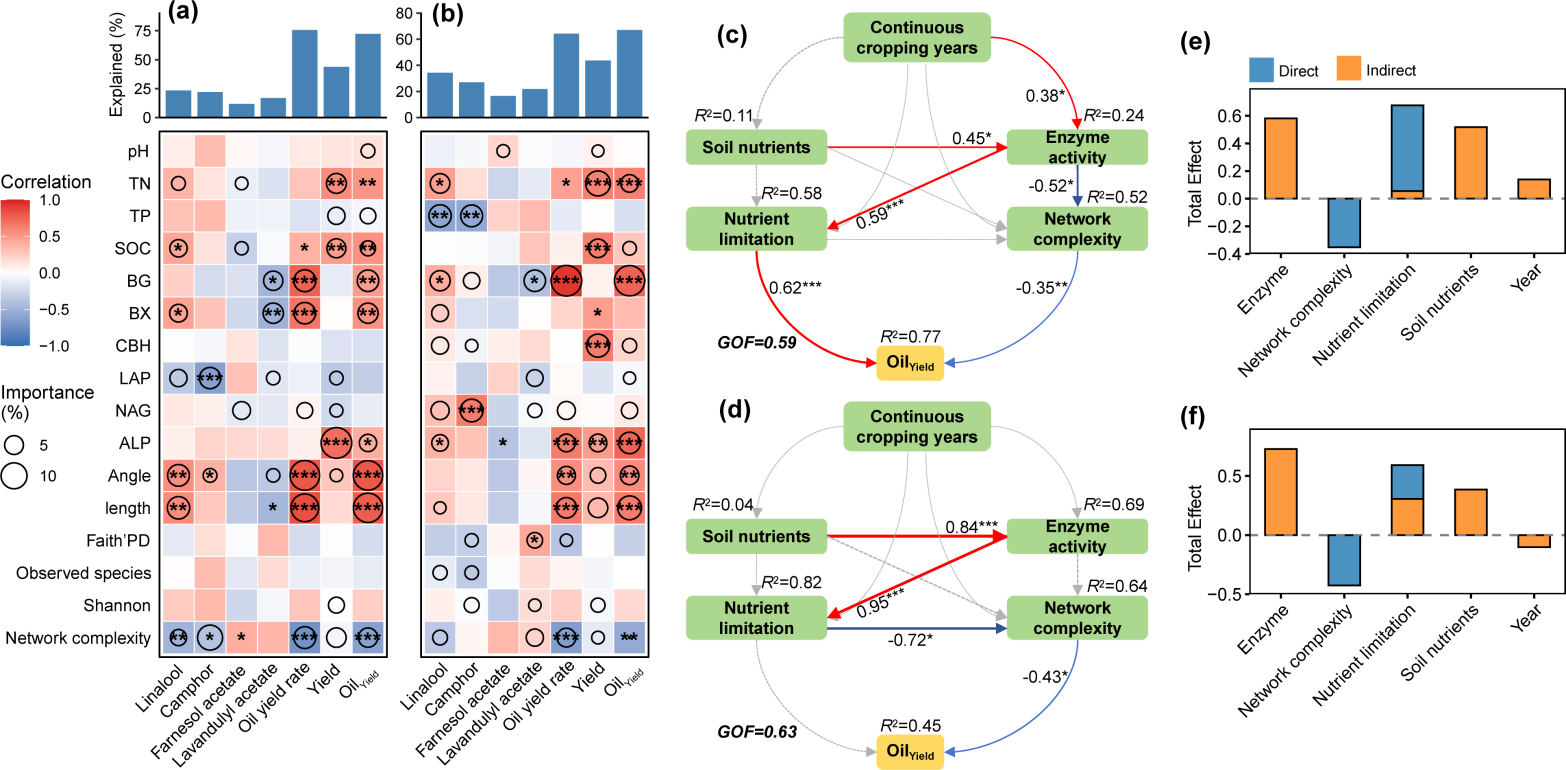
Multifactor contributions of essential oil yield and main components based on Pearson correlation and random forest models **(a, b)** and PLS-PM models **(c-f).** Panels **(a, c, e)** and **(b, d ,f)** correspond to the 0–20 cm and 20–40 cm soil layers, respectively. The upper bar charts indicate the total variance explanation rate of all explanatory variables for the target variable in the random forest model, while the lower heatmaps show the correlations between target variables and explanatory variables. In the heatmaps, the size of the circles represents the importance of each feature variable in the random forest model. *, **, and *** denote significant differences at the *P* < 0.05, *P* < 0.01, and *P* < 0.001 levels, respectively. Gray dashed lines indicate no significant effect, red lines represent significant positive effects, and blue lines represent significant negative effects, with the line thickness being proportional to the path coefficient. Soil nutrients: SOC, TN, and TP; Enzyme activity: BG, CBH, BX, NAG, LAP, and AP; Nutrient limitation: Vector length and Vector angle. *R*² represents the proportion of variance in each response variable that can be explained.

Correlation heatmaps incorporating variable importance revealed linkages among soil nutrients, enzyme activities, nutrient limitations, bacterial communities, and essential oil metrics (**Figures 7a, b**). Across both soil layers, nutrient limitation and enzyme activities (e.g., BG, BX) showed significant positive correlations with oil yield and extraction rate (*P<* 0.05). In contrast, microbial diversity indices (Faith’s PD, Observed species) exhibited weak associations with oil constituents, while co-occurrence network complexity negatively correlated with yield metrics (*P<* 0.05). Random forest importance analysis identified nutrient limitation as the primary driver of oil yield in the 0–20 cm layer, whereas BG activity and nutrient limitation dominated in the 20–40 cm layer. These factors collectively explained<40% of variance in key oil components (e.g., linalool, camphor), indicating limited predictive power for compositional shifts. Critically, soil C-cycling enzymes, nutrient limitation, and microbial network complexity emerged as key regulators of oil yield and quality. The PLS-PM demonstrated that in the 0–20 cm layer (Model C) (**Figures 7c, e**), monoculture duration directly influenced enzyme activity (r= 0.38, *P<* 0.01), subsequently regulating network complexity (r= –0.24, *P<* 0.05) and nutrient limitation (r= 0.50, *P<* 0.01), ultimately modulating oil yield (77% variance explained; GOF = 0.59). The 20–40 cm layer followed analogous pathways (45% variance explained; GOF = 0.63), confirming consistent mechanistic drivers across soil depths (**Figures 7d, f**).

## 4 Discussion

### 4.1 Long-term monoculture enhances essential oil yield without compromising quality

Contrary to the conventional understanding that continuous cropping typically leads to declines in both crop yield and quality (Chen et al., 2025; Zhao et al., 2024), this study reveals that perennial aromatic plants exhibit unique adaptability to long-term monoculture, a finding that refutes our initial hypothesis (Hypothesis 1). While essential oil yield continues to increase, the major constituents (e.g., linalool and camphor) remain stable; this compositional stability may enable the sustained release of allelopathic substances, thereby suppressing the growth of competing plant species (Zheljazkov et al., 2021). Furthermore, during long-term continuous cropping, beneficial microorganisms with functions such as phosphate solubilization, nitrogen fixation, and secretion of growth-promoting substances gradually become dominant taxa within the soil microbial community, facilitating more efficient nutrient uptake and utilization under conditions of limited soil fertility (Xugela Habuding et al., 2026). Collectively, these mechanisms form the basis for the competitive advantage maintained by lavender in long-term continuous cropping systems.

Lavender harvesting is limited to the floral stems, leaving the underground root system intact. This non-destructive harvesting approach allows the root system to be retained and continuously developed year after year, thereby progressively establishing and expanding its structural architecture (Thorup-Kristensen et al., 2020; Zhang et al., 2023). Studies have shown that the root system of lavender becomes increasingly developed with prolonged cultivation years (Tunc and von Wirén, 2025). The expanded root system enables more efficient exploration of soil space, facilitating the uptake of water and nutrients, thereby supporting sustained plant growth and essential oil yield (Fichtl et al., 2024). Furthermore, the continuous cultivation of perennial crops avoids the disruption of soil structure caused by annual tillage, contributing to the maintenance of soil aggregate stability and the reduction of nutrient loss (Zhang et al., 2023). The retention of the root system favors the stabilization of the rhizosphere microenvironment, thereby promoting the sustainable succession of rhizosphere microbial communities (Pang and Xu, 2024). This stability is closely associated with the synergistic effects among microbial functions (Hu et al., 2025). However, owing to the inherent life cycle of the root system (Tunc and von Wirén, 2025), the yield follows a unimodal trajectory—peaking at 3–6 years—followed by a subsequent decline attributable to root senescence, as observed in our study.

Regional heterogeneity analysis further elucidated the critical role of soil carbon pools in this process. The higher initial organic carbon content in HC provided a more robust carbon substrate availability for essential oil biosynthesis, aligning with the significant correlation between oil extraction rate and soil organic carbon (**Figure 7**). This confirms the central role of carbon supply in yield enhancement under continuous cropping (Ma et al., 2023). The gradual deceleration of yield growth may be attributed to resource constraints that limit the positive effects of continuous cropping (Ling et al., 2025). Studies have shown that when the rate of soil carbon and nitrogen depletion exceeds the rate of natural replenishment, the adaptive advantages conferred by continuous cropping may diminish (Pravia et al., 2019).

### 4.2 Dynamic effects of continuous cropping on soil physicochemical properties and enzyme activities

Conventional wisdom holds that continuous cropping leads to progressive soil nutrient depletion (Yu et al., 2024; Zheng et al., 2024), and our Hypothesis 2 was formulated based on this premise. However, during the continuous cropping of lavender, soil nutrient content actually exhibits a “low–high–low” trajectory, which clearly deviates from this expectation. By the third year of cultivation, nutrient levels increase, primarily because the continuous cultivation of perennial crops reduces soil disturbance, thereby mitigating tillage-induced nutrient loss (Audu et al., 2022; Yang et al., 2024c). Concurrently, the sustained secretion of organic compounds from plant roots, coupled with the gradual accumulation of unharvested stems and leaves in the shallow soil layer, provides a continuous input of organic materials. These inputs serve as abundant substrates for soil microorganisms and are subsequently mineralized into organic matter, thereby reducing nutrient leaching (Angst et al., 2025; Zeng et al., 2024a, 2024). The findings of this study indicate that by the sixth year of continuous cropping, nutrient content declines once again, suggesting that the effects of resource depletion induced by continuous cropping begin to manifest (Wang et al., 2024). Nevertheless, at this stage, soil nutrient levels remain above their initial values, indicating that sufficient nutrient reserves still exist to support plant growth and maintain, to a certain extent, the potential for sustained yield enhancement.

The carbon cycle-related enzymes, including BG and CBH, which are involved in cellulose degradation, and BX, which participates in hemicellulose degradation (Jian et al., 2016), exhibited a gradually increasing trend with planting years (**Figure 3**). This pattern may reveal the underlying mechanisms driving changes in soil carbon content. Studies have indicated that the increased secretion of carbon-degrading enzymes represents an adaptive response of microorganisms to carbon limitation (Sui et al., 2025), serving to accelerate the decomposition of organic carbon and thereby maintain ecosystem carbon balance (Feng et al., 2023). Such regulation of enzyme activity enables plants to efficiently acquire carbon even under conditions of limited carbon input, which may partly account for the sustained increase in essential oil yield (Zhang et al., 2022).

LAP and NAG are both enzymes involved in the nitrogen cycle, playing a role in nitrogen mineralization (Chen et al., 2023). The fluctuations in the activities of these nitrogen-related enzymes reflect the continuous adjustment of microbial strategies in response to changes in nutrient limitation, thereby aligning with the nitrogen demands of plants (Liu et al., 2023a). Specifically, when soil organic carbon and total nitrogen contents are relatively low, the activities of LAP and NAG increase significantly. This phenomenon aligns with the “resource allocation model” (Sinsabaugh et al., 2009), which posits that when available nitrogen in the soil is insufficient, microorganisms upregulate the secretion of nitrogen-acquiring enzymes (LAP and NAG) to mineralize organic nitrogen, thereby meeting their own nitrogen requirements as well as those of the plants. This elevation in enzyme activity represents an adaptive strategy employed by microorganisms under nitrogen-limited conditions. Furthermore, lavender, a plant with a high nitrogen demand, may selectively enrich ammonia-oxidizing bacteria through root exudates, thereby enhancing nitrogen transformation efficiency and alleviating nitrogen limitation (Hao et al., 2022). The activity of ALP exhibited considerable variability across multiple treatments, particularly in the 20–40 cm soil layer (**Figure 3**), and showed a clear negative correlation with soil available phosphorus content. When soil available phosphorus was at a relatively low level, ALP activity was significantly upregulated. This finding is consistent with the results reported by Allison and Vitousek (2005).

### 4.3 Stratified impacts of continuous cropping on soil bacterial communities

This study found that the response of soil bacterial communities to continuous cropping exhibited distinct stratification, which did not fully align with our Hypothesis 3. Specifically, the α-diversity of bacteria in the surface soil (0–20 cm) was not significantly affected by the duration of continuous cropping, whereas it significantly decreased with increasing cropping years in the subsurface soil (20–40 cm). This stratification effect is closely related to the heterogeneity of the soil environment (He et al., 2023). The surface soil, directly influenced by tillage disturbance, plant litter input, and active root exudation (Huang et al., 2023), likely harbors a microbial community with higher species turnover rates and functional redundancy. This community may exhibit stronger adaptability or faster succession rates in response to persistent chemical pressures induced by continuous cropping, such as the accumulation of specific root exudates (Peng et al., 2025). In contrast, the physicochemical properties of the deeper soil environment are relatively stable, and its microbial community structure is more conservative. It may be more sensitive to systemic changes induced by continuous cropping (Peng et al., 2025). Once the original equilibrium is disrupted, its resilience is weaker, leading to a sustained decline in diversity (He et al., 2025). This finding aligns with the general conclusion that continuous cropping reduces microbial diversity (Song et al., 2025; Zhang et al., 2025), but clarifies that its impact is uneven across the soil profile, emphasizing the need to consider soil depth when assessing continuous cropping effects. At the phylum level, increasing continuous cropping duration led to a consistent declining trend in the relative abundance of *Proteobacteria* across both soil layers. This change is closely linked to the dynamics of soil carbon and nitrogen nutrients under continuous cropping. As a typical copiotrophic group, the decline in Proteobacteria abundance may reflect the phased changes in the supply of readily available soil nutrients under long-term continuous cropping (Yang et al., 2025). Particularly, the resource pressure from nutrient decline beginning at the 6-year continuous cropping stage likely limited the proliferation of such high-nutrient-demanding microorganisms (Baker et al., 2024; Li et al., 2022). Conversely, the accumulation of *Verrucomicrobiota* may be related to its oligotrophic characteristics and adaptability to specific stresses, reflecting the selective pressure of the altered soil microenvironment under continuous cropping.

Although the α-diversity of surface soil bacteria remained stable, the complexity of the bacterial co-occurrence network significantly decreased across the entire soil profile with increasing continuous cropping duration. This reveals a fundamental restructuring of microbial ecological interaction strategies driven by continuous cropping (Na et al., 2021). Traditionally, a decline in network complexity is often regarded as a sign of ecosystem degradation and functional instability (Li et al., 2023; Wu et al., 2024). However, in this study, network simplification did not show a widespread negative correlation with essential yield (Zhang et al., 2025; Li et al., 2026). Instead, under specific conditions (e.g., three years of continuous cropping), it even coexisted with higher yields. We propose that this reflects a shift in the microbial community’s strategy under sustained, specific host selection pressure—from a robust strategy reliant on extensive functional redundancy to an efficiency strategy characterized by high functional specialization (Li et al., 2023; Wu et al., 2024). Long-term continuous cropping may have filtered and enriched microbial taxa capable of adapting to this specific chemical microenvironment (Zhang et al., 2025). While this led to an overall decrease in connection numbers and functional redundancy, these retained taxa may have formed extremely efficient and direct functional pathways among themselves or internally to support the host’s core needs (such as nutrient acquisition) (Robinson and Strauss, 2020). This could be sufficient to maintain or even optimize plant physiological metabolism at specific stages, thus not manifesting negative impacts on yield. Nevertheless, the widespread decline in network complexity implies that the soil microbial community’s buffering capacity against external disturbances is weakened (Qiao et al., 2024). This mechanism also helps explain the discrepancy between our findings and those from most studies on annual crops. For systems like soybean and peanut, continuous cropping obstacles often erupt and become the dominant limiting factor within a relatively short period (5–13 years) (Zhang et al., 2025; Li et al., 2026). Therefore, maintaining a highly complex microbial network is crucial for ensuring immediate and comprehensive stress resistance functions; network simplification in these systems is often directly linked to functional impairment and yield reduction (Ma et al., 2024). However, in perennial lavender systems, the accumulation and manifestation of ecological stress may be a slower process.

### 4.4 Mechanistic insights into microbial regulation of essential oil yield

The PLS-PM model established a direct link between microbial processes and plant productivity under continuous cropping conditions. Microbial carbon limitation was positively correlated with oil yield, reflecting a synergistic carbon allocation mechanism (Zhuang et al., 2024). Specifically, microbial competition for carbon may transmit signals to plants, prompting them to enhance photosynthetic efficiency or accelerate organic matter mineralization (Zhuang et al., 2024), thereby providing more bioavailable carbon for terpenoid biosynthesis (Singh et al., 2025). Meanwhile, the alleviation of nitrogen limitation was positively associated with oil yield, confirming the importance of nitrogen supply for essential oil synthesis (Ordóñez et al., 2025). As a key component of enzymes and chlorophyll, adequate nitrogen not only supports metabolic enzyme activity but also ensures normal photosynthetic function (Liu et al., 2025c). Notably, bacterial network complexity was negatively correlated with oil yield, a finding that challenges the conventional notion that “higher ecosystem complexity leads to higher productivity” (Ma et al., 2024), reflecting a nonlinear relationship between microbial community structure and plant productivity under long-term continuous cropping. A plausible interpretation of this phenomenon is that a decline in bacterial network complexity does not necessarily equate to immediate deterioration of soil health, but rather indicates an increase in ecological risk (Qiao et al., 2024). The reduction in network nodes and connectivity induced by continuous cropping essentially reflects a gradual decline in the buffering capacity of the soil microbial community in response to environmental disturbances (Zhang et al., 2024; Liu et al., 2020). For instance, when faced with disturbances such as extreme climatic events, pathogen invasion, or drastic fluctuations in soil nutrients, the simplified network, lacking sufficient functional redundancy, struggles to maintain ecological stability (Zhang et al., 2024), which may ultimately lead to a significant decrease in essential oil yield. Therefore, although network simplification exhibited a significant positive effect on yield in the present study, this effect may be temporary. Short-term monoculture may meet immediate yield demands to some extent, whereas long-term continuous cropping may introduce a series of sustained and cumulative ecological challenges.

Regional analysis showed that the soil bacterial network degradation rate in the HC site was relatively slow, which may be closely related to its higher initial soil fertility. This finding aligns with the research conclusion proposed by Qiao, that high-quality soil can reduce the sensitivity of crop yield to environmental changes (Qiao et al., 2022). Collectively, our findings reveal a dual dynamic, characterized by the coexistence of nutrient-driven yield enhancement along with network-mediated vulnerability, thereby providing a theoretical framework for balancing short-term productivity and long-term sustainability in the management of aromatic crops.

## 5 Conclusions

This study investigated the effects of long-term continuous cropping (1-, 3-, and 6-year durations) on *lavender* essential oil production, soil properties, enzyme activities, and bacterial communities in Xinjiang’s Ili River Valley. Key findings show that continuous cropping preserved the stability of signature oil constituents (e.g., linalool, camphor) while significantly enhancing oil yield—a stark contrast to typical monoculture yield-quality trade-offs. This unique response stems from perennial cultivation practices: partial biomass retention during harvest and minimized tillage sustain soil structure. Soil nutrients exhibited a triphasic “low-high-low” dynamic, peaking at 3 years due to reduced disturbance, cumulative litter inputs, and root exudate-mediated nutrient priming. Sustained increases in C-cycling enzyme activities supported carbon availability during later stages. Bacterial responses showed depth-dependent stratification: α-diversity remained stable in surface soils (0–20 cm) but declined significantly in subsurface layers (20–40 cm). Crucially, co-occurrence network complexity decreased across both depths, evidenced by reduced nodes and edges. Microbial regulation featured synergistic carbon limitation intensification and nitrogen limitation alleviation, driving yield gains, while network simplification posed long-term resilience risks. As the first systematic exploration of *lavender*’s “stable quality, enhanced yield” monoculture effect, this work redefines sustainable aromatic crop management. We recommend a 3–5 year monoculture rotation with leguminous green manure, combined with functional microbial inoculants and no-till practices, to balance short-term productivity gains with long-term soil ecosystem health for the Ili Valley *lavender* industry, grounded in 3-year nutrient peaks and long-term microbial network risks.

## Data Availability

All data supporting the results of this study are included in the article, and datasets are publicly available on the Github (https://github.com/Zengjia1998/DATA1.git).
