# Supplementary material for: Continuous cropping of lavender (Lavandula angustifolia Mill.) enhances essential oil yield without compromising quality
Source: Front Plant Sci. 2026 May 29;17:1840314. doi: 10.3389/fpls.2026.1840314 (PMC13259690; doi:10.3389/fpls.2026.1840314)
Supplement: Supplementary file 1 [file SupplementaryFile1.docx]

**Continuous cropping of *lavender* enhances essential oil yield without compromising quality**

**This file includes:**

**● Supplementary Figures S1-S6.**

**● Supplementary Text S1**

**● Supplementary Table S1-S3**


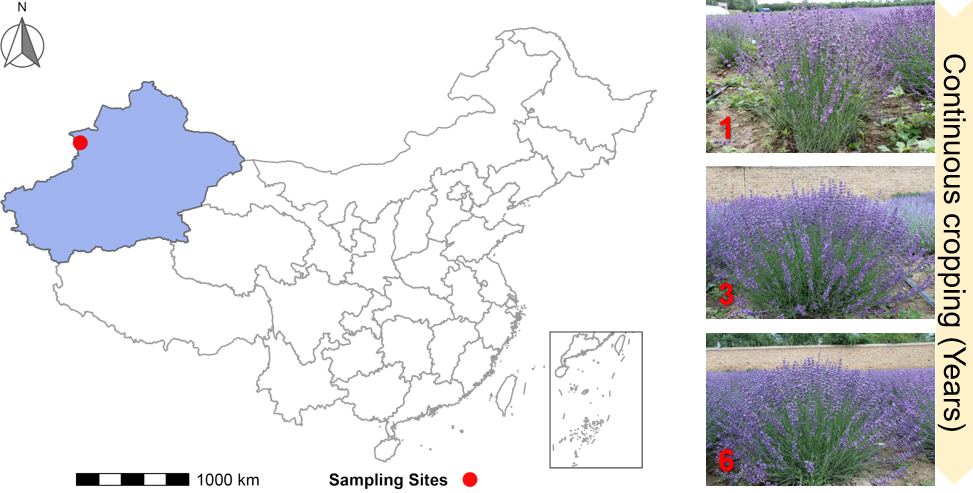


**Figure S1. Sampling Location Information**


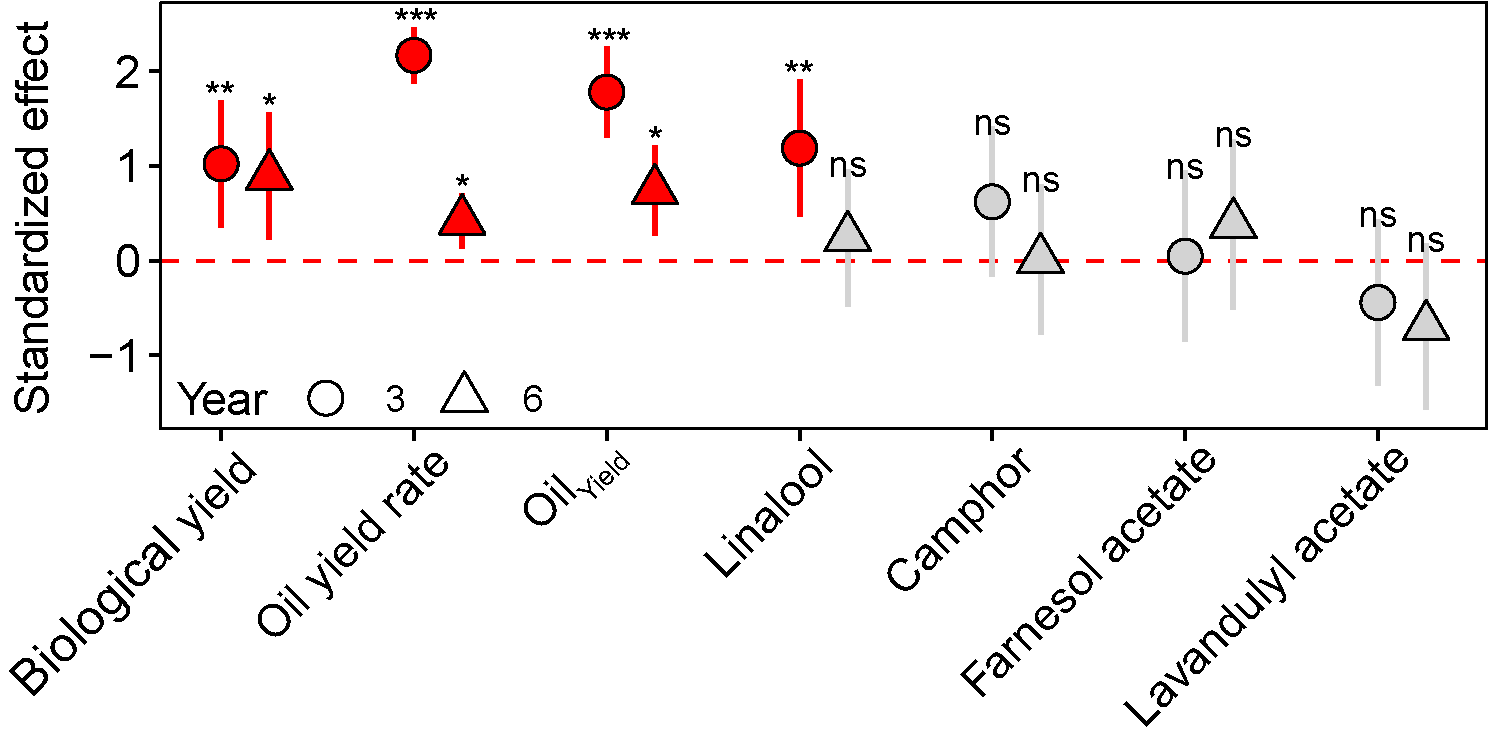


**Figure S2. The linear mixed-effects model demonstrates the influence of the sampling year on the yield and quality of lavender and its essential oil.** It shows that the sampling area is considered as a random effect, while the consecutive cropping years are regarded as a fixed effect. The standardized effect size (points) of consecutive cropping on the target variable and its 95% confidence interval (error lines) are analyzed. ns indicates *p* > 0.05, not significantly different. *, **, *** represent significant differences at *p* < 0.05, < 0.01, < 0.001 levels, respectively.


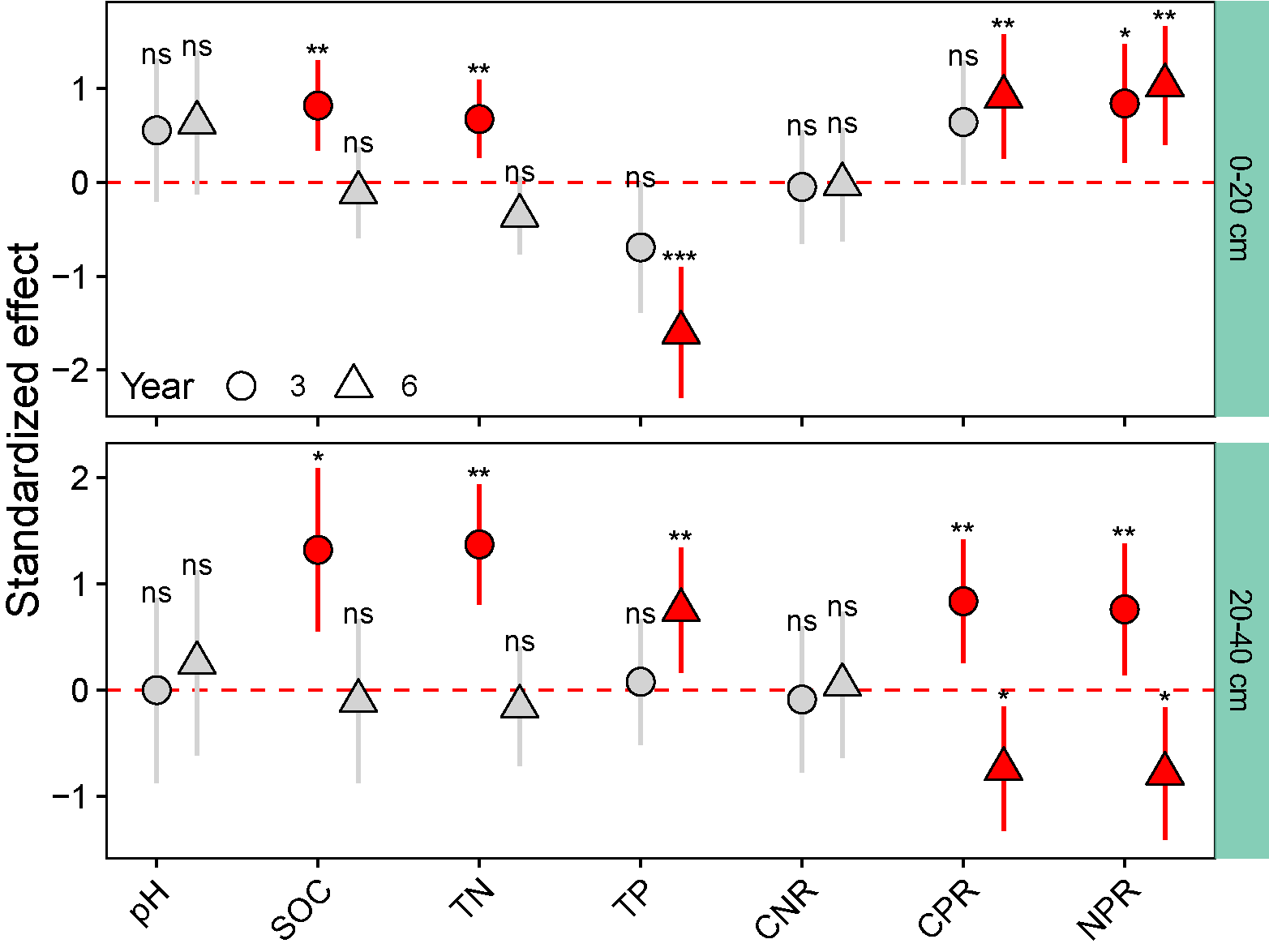


**Figure S3. The linear mixed-effects model demonstrates the influence of the sampling years on the physical and chemical properties of the soil.** It shows that the sampling area is considered as a random effect, while the consecutive cropping years are regarded as a fixed effect. The standardized effect size (points) of consecutive cropping on the target variable and its 95% confidence interval (error lines) are analyzed. ns indicates *p* > 0.05, not significantly different. *, **, *** represent significant differences at *p* < 0.05, < 0.01, < 0.001 levels, respectively.


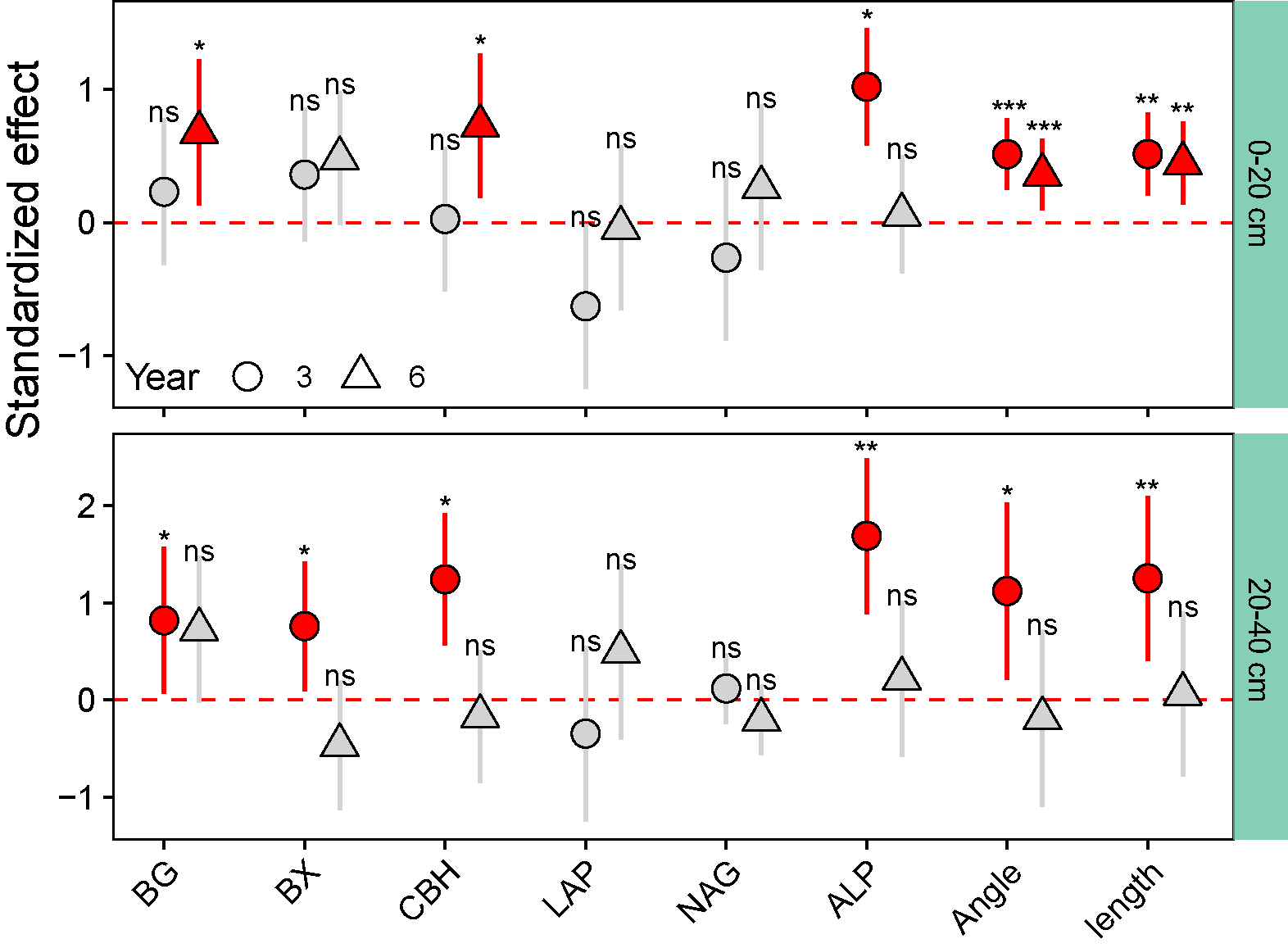


**Figure S4. The linear mixed-effects model demonstrates the influence of the sampling years on soil enzyme activity and nutrient limitation.** It shows that the sampling area is considered as a random effect, while the consecutive cropping years are regarded as a fixed effect. The standardized effect size (points) of consecutive cropping on the target variable and its 95% confidence interval (error lines) are analyzed. ns indicates *p* > 0.05, not significantly different. *, **, *** represent significant differences at *p* < 0.05, < 0.01, < 0.001 levels, respectively.


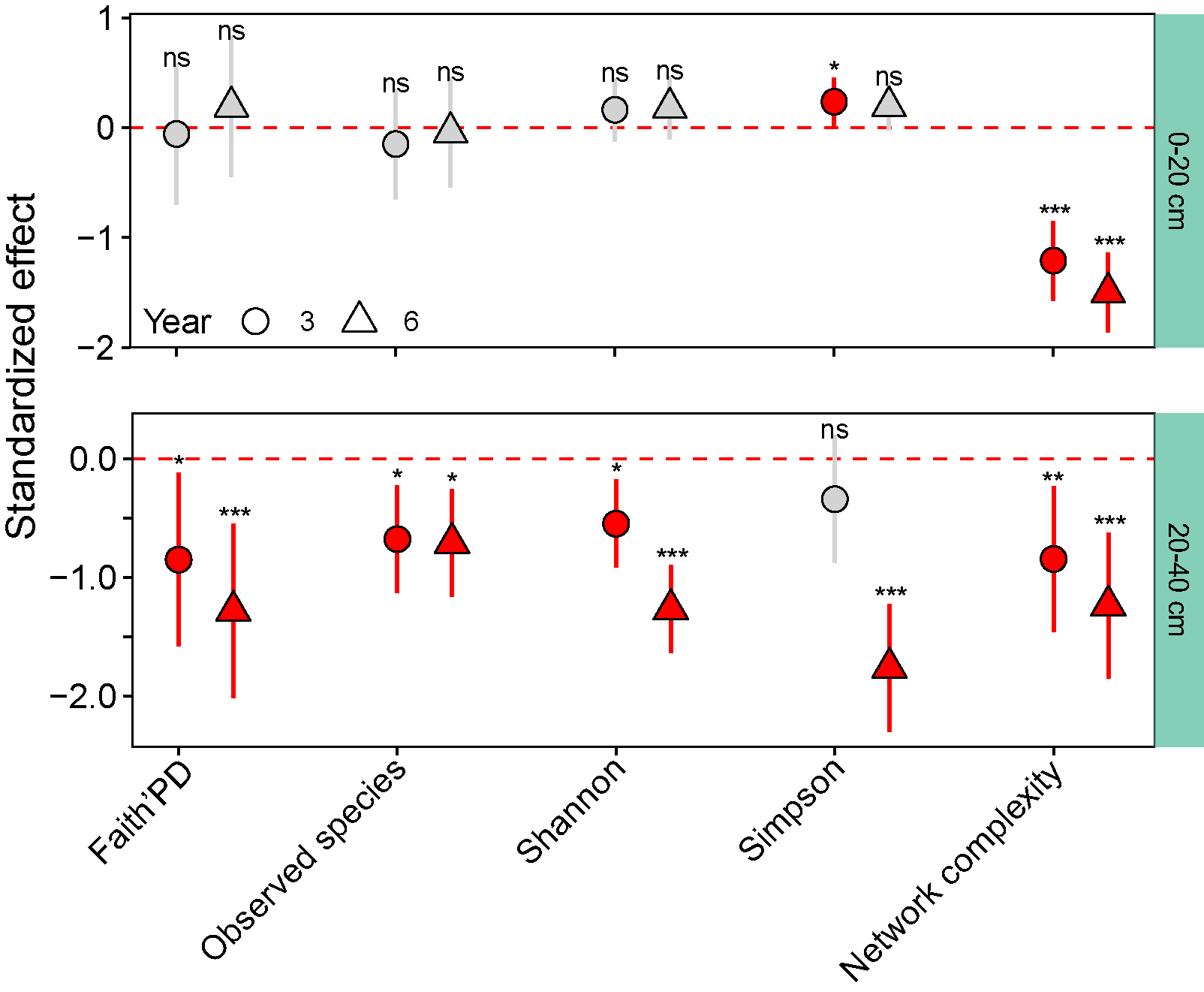


**Figure S5. The linear mixed-effects model demonstrates the influence of the sampling years on the soil microbial community.** It shows that the sampling area is considered as a random effect, while the consecutive cropping years are regarded as a fixed effect. The standardized effect size (points) of consecutive cropping on the target variable and its 95% confidence interval (error lines) are analyzed. ns indicates *p* > 0.05, not significantly different. *, **, *** represent significant differences at *p* < 0.05, < 0.01, < 0.001 levels, respectively.


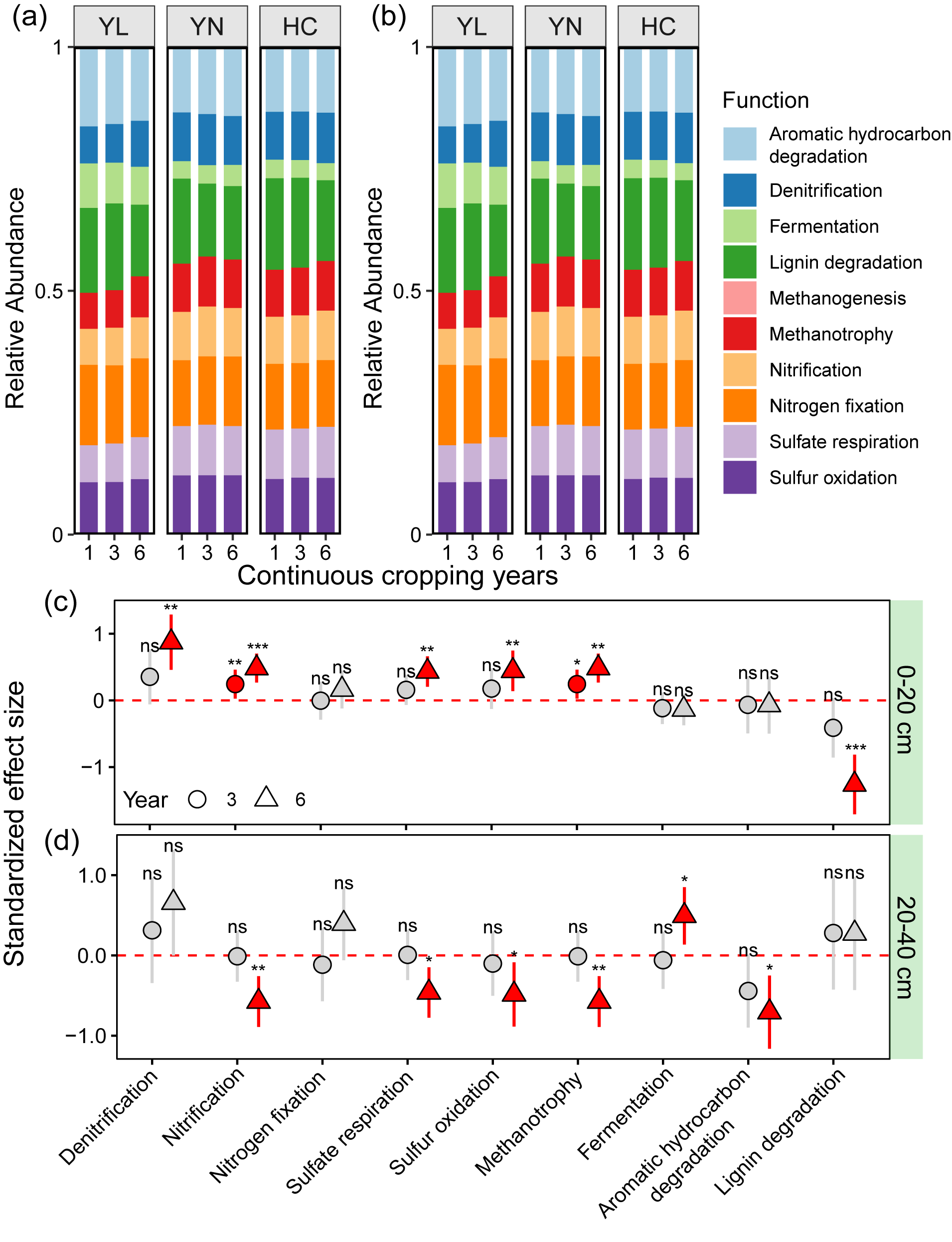


**Figure S6. The impact of intercropping on the functional properties of soil bacterial communities.** (ab) Bar charts respectively illustrate the cumulative effects of intercropping on the functional characteristics of soil bacterial communities in the 0-20 and 20-40 cm soil layers;（cd）It shows that the sampling area is considered as a random effect, while the consecutive cropping years are regarded as a fixed effect. The standardized effect size (points) of consecutive cropping on the target variable and its 95% confidence interval (error lines) are analyzed. ns indicates *p* > 0.05, not significantly different. *, **, *** represent significant differences at *p* < 0.05, < 0.01, < 0.001 levels, respectively.

**Text S1 Enzyme activity determination.** The activity of C-acquisition enzyme (BG: β-1,4-glucosidase, CBH: Cellobiohydrolase, BX: β-xylosidase), N-acquisition enzyme (NAG: β-1,4-N-acetylglucosaminidase and LAP: L-leucine aminopeptidase), and organic P-acquisition enzyme (AP: alkaline phosphatase) were determined following modified methods of standard fluorometric techniques (Saiya-Cork *et al*., 2002; Sinsabaugh *et al*., 2008). Briefly, the analysis included eight replicate wells for each sample, blank, negative control, quench standard and reference standard in a 96-well plate. Sample suspensions were prepared by adding 1 g fresh soil to 125 mL of 50 mM acetate buffer with pH 8.5 and homogenizing. 50 μL of 200 mM substrate solution and 200 μL of sample suspension were added into sample wells. 50 μL of acetate buffer and 200 μL of sample suspension were added into blank wells. 50 μL of mM substrate solution and 200 μL of acetate buffer were added into negative control wells. 50 μL of standard and 200 μL sample suspension were added into quench standard wells. 50 μL of standard 200 μL of acetate buffer were added into reference standard wells. The microplates were incubated in the dark at 25 °C for 4 hours. And then 10 μL of 0.5 mol·L^-1^ NaOH was added to each well to stop the reaction at the end of incubation. Finally, fluorescence was measured using a microplate reader (Tecan Infinite M200, Salzburg, Austria) at 365 nm excitation and 450 nm emission. According to fluorescence values, soil absolute enzyme activity was expressed as nmol·h^−1^·g^−1^ dry soil.

**References:**

Saiya-Cork, K.R., R.L. Sinsabaugh, D.R.J.S.B. Zak, and Biochemistry. 2002. The effects of long term nitrogen deposition on extracellular enzyme activity in an Acer saccharum forest soil. *Soil Biology and Biochemistry*. 34:1309-1315.

Sinsabaugh, R.L., C.L. Lauber, M.N. Weintraub, B. Ahmed, S.D. Allison, C. Crenshaw, A.R. Contosta, D. Cusack, S. Frey, M.E. Gallo, T.B. Gartner, S.E. Hobbie, K. Holland, B.L. Keeler, J.S. Powers, M. Stursova, C. Takacs-Vesbach, M.P. Waldrop, M.D. Wallenstein, D.R. Zak, and L.H. Zeglin. 2008. Stoichiometry of soil enzyme activity at global scale. *Ecology Letters*. 11:1252-1264.

**Table S1. Effects of continuous cropping duration on soil physical and chemical indicators.**

|  | **Soil depth** | **Years** | **HC** | **YL** | **YN** | **Two-Way ANOVA** |
| --- | --- | --- | --- | --- | --- | --- |
| **pH** | 0-20 cm | 1 | 7.83±0.13 a | 7.97±0.13 a | 7.57±0.13 a | site * |
|  |  | 3 | 7.70±0.13 a | 8.17±0.13 a | 7.93±0.13 a | Years ns |
|  |  | 6 | 7.83±0.13 a | 8.10±0.13 a | 7.93±0.13 a | site:Years ns |
|  | 20-40 cm | 1 | 8.10±0.16 a | 8.03±0.16 a | 7.87±0.16 a | site ns |
|  |  | 3 | 8.13±0.16 a | 7.97±0.16 a | 7.90±0.16 a | Years ns |
|  |  | 6 | 7.90±0.16 a | 8.17±0.16 a | 8.13±0.16 a | site:Years ns |
| **CNR** | 0-20 cm | 1 | 7.85±0.42 b | 5.46±0.42 a | 4.72±0.42 a | site ** |
|  |  | 3 | 5.36±0.42 a | 6.61±0.42 a | 5.85±0.42 a | Years ns |
|  |  | 6 | 6.50±0.42 ab | 6.65±0.42 a | 4.80±0.42 a | site:Years ** |
|  | 20-40 cm | 1 | 9.03±0.53 a | 6.25±0.53 a | 8.53±0.53 a | site * |
|  |  | 3 | 7.21±0.53 a | 7.19±0.53 ab | 9.01±0.53 a | Years ns |
|  |  | 6 | 7.32±0.53 a | 8.58±0.53 b | 8.14±0.53 a | site:Years * |
| **CPR** | 0-20 cm | 1 | 6.36±0.37 a | 4.85±0.37 a | 3.58±0.37 a | site ** |
|  |  | 3 | 5.15±0.37 a | 5.74±0.37 a | 8.36±0.37 b | Years *** |
|  |  | 6 | 8.58±0.37 b | 8.39±0.37 b | 4.15±0.37 a | site:Years *** |
|  | 20-40 cm | 1 | 9.05±0.43 b | 8.85±0.43 b | 5.42±0.43 a | site *** |
|  |  | 3 | 7.84±0.43 ab | 13.14±0.43 c | 8.13±0.43 b | Years *** |
|  |  | 6 | 6.26±0.43 a | 6.05±0.43 a | 5.86±0.43 a | site:Years *** |

Note: Results of two-way ANOVA demonstrate the effects of Sampling Site (Site), Continuous Cropping Duration (Years), and their Interaction (Site × Years). ns indicates *P* > 0.05, not significantly different. *, **, *** represent significant differences at *P* < 0.05, < 0.01, < 0.001 levels, respectively. pH: Soil acidity-alkalinity; CNR: Soil carbon-nitrogen ratio; CPR: Soil carbon-phosphorus ratio.

**Table S2 Effects of continuous lavender cropping duration on bacterial co-occurrence network topology.**

| **Network topology characteristics** | **Soil depth** | **Years** | **HC** | **YL** | **YN** | **Two-Way ANOVA** |
| --- | --- | --- | --- | --- | --- | --- |
| Clustering coefficient | 0-20 cm | 1 | 0.444±0.005 a | 0.407±0.005 a | 0.472±0.005 a | site *** |
|  |  | 3 | 0.468±0.005 b | 0.434±0.005 b | 0.464±0.005 a | Years ** |
|  |  | 6 | 0.445±0.005 a | 0.441±0.005 b | 0.467±0.005 a | site:Years ** |
|  | 20-40 cm | 1 | 0.459±0.005 ab | 0.412±0.005 a | 0.456±0.005 a | site *** |
|  |  | 3 | 0.448±0.005 a | 0.433±0.005 b | 0.476±0.005 b | Years * |
|  |  | 6 | 0.468±0.005 b | 0.437±0.005 b | 0.462±0.005 ab | site:Years * |
| Degree | 0-20 cm | 1 | 6.59±0.20 b | 6.08±0.20 a | 5.91±0.20 a | site ns |
|  |  | 3 | 5.81±0.20 a | 6.20±0.20 a | 6.23±0.20 a | Years ns |
|  |  | 6 | 6.04±0.20 ab | 6.47±0.20 a | 6.58±0.20 a | site:Years * |
|  | 20-40 cm | 1 | 6.09±0.21 a | 6.59±0.21 a | 6.28±0.21 b | site ** |
|  |  | 3 | 6.40±0.21 a | 6.50±0.21 a | 5.28±0.21 a | Years ns |
|  |  | 6 | 6.44±0.21 a | 6.34±0.21 a | 6.11±0.21 b | site:Years * |
| Network density | 0-20 cm | 1 | 0.024±0.001 b | 0.020±0.001 a | 0.022±0.001 ab | site * |
|  |  | 3 | 0.021±0.001 ab | 0.020±0.001 a | 0.021±0.001 a | Years ns |
|  |  | 6 | 0.020±0.001 a | 0.020±0.001 a | 0.024±0.001 b | site:Years * |
|  | 20-40 cm | 1 | 0.023±0.001 a | 0.022±0.001 a | 0.024±0.001 b | site ** |
|  |  | 3 | 0.026±0.001 ab | 0.023±0.001 a | 0.018±0.001 a | Years ** |
|  |  | 6 | 0.027±0.001 b | 0.022±0.001 a | 0.026±0.001 b | site:Years *** |
| Average path length | 0-20 cm | 1 | 3.57±0.06 a | 3.82±0.06 a | 3.81±0.06 b | site ns |
|  |  | 3 | 3.76±0.06 ab | 3.75±0.06 a | 3.69±0.06 ab | Years ns |
|  |  | 6 | 3.90±0.06 b | 3.74±0.06 a | 3.56±0.06 a | site:Years ** |
|  | 20-40 cm | 1 | 3.59±0.06 a | 3.77±0.06 a | 3.70±0.06 a | site ** |
|  |  | 3 | 3.47±0.06 a | 3.71±0.06 a | 3.79±0.06 a | Years ns |
|  |  | 6 | 3.64±0.06 a | 3.60±0.06 a | 3.79±0.06 a | site:Years ns |
| Betweenness centralization | 0-20 cm | 1 | 0.052±0.005 a | 0.037±0.005 a | 0.041±0.005 a | site ** |
|  |  | 3 | 0.040±0.005 a | 0.037±0.005 a | 0.056±0.005 ab | Years ns |
|  |  | 6 | 0.057±0.005 a | 0.035±0.005 a | 0.065±0.005 b | site:Years * |
|  | 20-40 cm | 1 | 0.041±0.006 a | 0.047±0.006 a | 0.074±0.006 ab | site *** |
|  |  | 3 | 0.047±0.006 a | 0.052±0.006 a | 0.064±0.006 a | Years ns |
|  |  | 6 | 0.041±0.006 a | 0.042±0.006 a | 0.089±0.006 b | site:Years ns |
| Modularity | 0-20 cm | 1 | 0.454±0.013 a | 0.530±0.013 a | 0.428±0.013 b | site *** |
|  |  | 3 | 0.485±0.013 ab | 0.523±0.013 a | 0.402±0.013 ab | Years ns |
|  |  | 6 | 0.523±0.013 b | 0.505±0.013 a | 0.365±0.013 a | site:Years ** |
|  | 20-40 cm | 1 | 0.491±0.014 a | 0.511±0.014 a | 0.399±0.014 a | site *** |
|  |  | 3 | 0.457±0.014 a | 0.505±0.014 a | 0.407±0.014 a | Years ns |
|  |  | 6 | 0.487±0.014 a | 0.507±0.014 a | 0.411±0.014 a | site:Years ns |

Note: Results of two-way ANOVA demonstrate the effects of Sampling Site (Site), Continuous Cropping Duration (Years), and their Interaction (Site × Years). ns indicates *P* > 0.05, not significantly different. *, **, *** represent significant differences at *P* < 0.05, < 0.01, < 0.001 levels, respectively.

**Table S3. Overview of the study area.**

| **Sampling sites** | **Altitude/m** | **Precipitation/mm** | | **Soil Water Content/%** | **soil type (FAO classification)** |
| --- | --- | --- | --- | --- | --- |
| **YL** | 670 | April | 24.17 | 15.67 | Grey calcareous soil |
|  |  | May | 46.8 |  |  |
|  |  | June | 22.47 |  |  |
|  |  | July | 33.17 |  |  |
| **YN** | 841 | April | 20.53 | 15.72 | Grey calcareous soil |
|  |  | May | 20.87 |  |  |
|  |  | June | 57.33 |  |  |
|  |  | July | 37.37 |  |  |
| **HC** | 691 | April | 9.33 | 13.67 | Grey calcareous soil |
|  |  | May | 29.83 |  |  |
|  |  | June | 35.83 |  |  |
|  |  | July | 28.3 |  |  |
